# Large zoom ratio and adaptive aberration correction microscope using 4DPSF-aware Physical Degradation-guided Network

**DOI:** 10.1038/s41377-025-02155-8

**Published:** 2026-03-03

**Authors:** Dong-Xu Yu, Zhao Jiang, Yi Zheng, Hao-Ran Zhang, Rong-Qiang Li, You-Ran Zhao, Xiao-Ke Lu, Yu-Cheng Lin, Chao Liu, Qiong-Hua Wang

**Affiliations:** 1 School of Instrumentation and Optoelectronic Engineering, Beihang University, Beijing, China; 2 School of Remote Sensing Science and Technology, Aerospace Information Technology University, Jinan, China

**Keywords:** Imaging and sensing, Microscopy

## Abstract

In response to the emerging demand for dynamic and cross-scale microscopic observation in fields such as biology, medicine, and materials science, the liquid lens has been widely adopted in modern microscopy to enable real-time, continuous optical zooming. However, the limited optical power of the liquid lens restricts the zoom range, and the nonlinear dynamic aberrations introduced during zooming can significantly degrade image quality. To address these challenges, a continuous optical zoom microscope with a large zoom ratio and adaptive aberration correction is proposed, based on an end-to-end joint optimization framework that integrates optical design and neural network guided by physical degradation. By incorporating spatially-variant, multi-wavelength, and continuous-magnification 4D PSF of the system as physical priors, this work achieves fast and high-quality continuous zoom imaging from 10.6× ~ 101.4×, while adaptively correcting complex aberrations that vary with both magnification and spatial location. The core hardware component of the system is a zoom objective lens with a movable relay image plane, which especially integrates electrowetting liquid lenses. On the algorithmic side, 4DPSF-aware Physical Degradation-guided Network (4DPSF-PDNet) is introduced for adaptive aberration correction during the zooming process. By embedding the PSF into the network, the method effectively suppresses distortions and artifacts and achieves precise correction of complex, dynamically varying aberrations. Experimental results demonstrate that the proposed adaptive continuous microscope holds significant promise for a wide range of applications in biology, medical diagnostics, and materials science.

## Abstract (web summary)

A continuous optical zoom microscope based on an end-to-end joint optimization framework with large zoom ratio and adaptive aberration correction is proposed.

## Graphical abstract

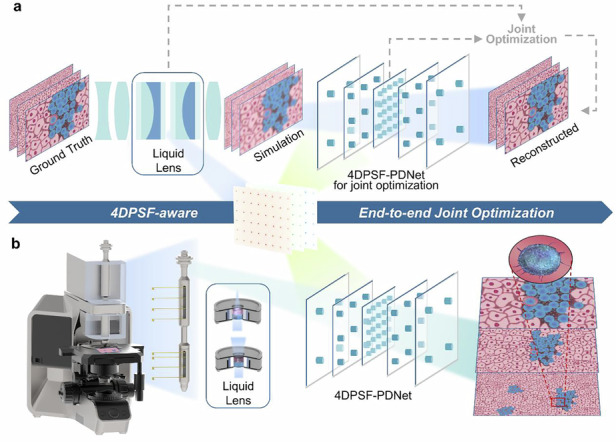

## Introduction

Optical microscopes are indispensable tools for exploring the microscopic world, playing a vital role in biomedicine, materials science, and industrial inspection^1–7^. Traditional microscopes rely on switching between objective lenses of different magnifications for zooming, which leads to discontinuous zoom, slow transition speed, and image jitter.

In recent years, emerging liquid lenses have been widely applied in display and imaging systems due to the advantages such as integration, adaptive zoom and low consumption^8–17^. In the exploration of microscopic imaging technology, researchers have primarily focused on inserting a single liquid lens into the optical path to extend the axial depth of field^18–24^. However, a single liquid lens is incapable of achieving zoom imaging in microscopy without mechanical actuation. Recent advancements in liquid lens technology enable adaptive electrical control of focal length to facilitate zoom imaging^25–29^. In 2016, a prototype microscope objective integrating four liquid lenses achieved a continuous zoom range with a zoom ratio of approximately 2×^25^. In 2022, a continuous zoom microscope with extended depth of field and 3D reconstruction capability was introduced, improving the zoom ratio to 6×, though further expansion remained challenging^29^. The focal power and aberration limitations of the liquid lens make it difficult to further increase the magnification ratio.

The advantage of optical systems based on liquid lens design is their capability to achieve cross-scale zooming functions. However, at varying zoom scales and within different spatial regions of the same scale, these systems are susceptible to diverse types of complex aberrations, which lead to the degradation of image quality during continuous zooming^29,30^. Researchers have turned to deep learning approaches for image quality enhancement, where incorporating optical priors has shown promise in improving reconstruction performance^31–39^. In 2021, a PSF-aware deep deconvolution network was proposed to enhance the imaging quality of simple lens^40^. In 2022, a two-stage method was introduced that first estimated the PSF for detail sharpening, followed by a convolutional neural network to eliminate lateral chromatic aberrations^41^. In the same year, researchers utilized PSF-generated simulated datasets for learnable signal-to-noise ratio (SNR) Wiener deconvolution to achieve denoising and detail restoration^42^. And due to the lack of physical prior knowledge, neural network methods that are trained only on paired images through supervised learning usually perform poorly on real data, resulting in limited adaptability and unsatisfactory results^43–45^. Moreover, these methods focus solely on post-processing image restoration, without integrating physical knowledge into the design of the optical system. As such, they fail to achieve optimal end-to-end performance. Therefore, there is an urgent need for a joint optimization framework that combines optical simulation and algorithm development guided by physical information.

In this work, an adaptive continuous microscope is proposed through a joint optimization framework that integrates end-to-end optical simulation with physics-informed neural networks. This paper's contributions are as follows:

1. **Physical degradation guided end-to-end joint optimization framework**. An end-to-end joint optimization framework is proposed, combining optical design and neural network under the guidance of physical degradation information. By introducing spatially varying, multi-wavelength, and continuous-magnification four-dimensional point spread function (4D PSF) as physical prior information, the proposed approach enables joint optimization of the optical simulation and neural network, significantly improving the continuous zoom range and imaging quality.
2. **Continuous high-quality imaging at a large zoom ratio**. A liquid lens-based continuous optical zoom microscope with a large zoom ratio is designed and integrated with the 4DPSF-aware Physical Degradation-guided Network (4DPSF-PDNet). The designed microscope achieves fast, continuous zoom imaging and adaptively corrects complex aberrations that vary with magnification levels and spatial regions.
3. **Physical priors guided a data-driven model with physics constraints**. A deep learning model guided by physical prior information, 4DPSF-PDNet, is developed. The model utilizes a data-driven, learnable Wiener filtering module in the frequency domain for initial noise suppression and spatial structure restoration. It can perform aberration correction and fine detail reconstruction through a degradation-guided multi-head self-attention mechanism. Additionally, a physics-constrained loss function is proposed to suppress non-physical artifacts and ensure more accurate restoration.

Overall, the proposed microscope achieves high-quality continuous zoom imaging across a magnification range of 10.6× ~ 101.4×. The proposed 4DPSF-PDNet outperforms other image aberration correction models, demonstrating superior performance in both visual quality and quantitative metrics. Compared with existing state-of-the-art physics-guided image reconstruction methods, it achieves 2.5 dB improvement in Peak Signal-to-Noise Ratio. The proposed microscope demonstrates the capability to achieve both large-range optical zoom and high-resolution imaging, showing great potential for applications in pathological diagnosis, cell detection, and material analysis.

## Results

### Structure and principle

**Fig. 1:**
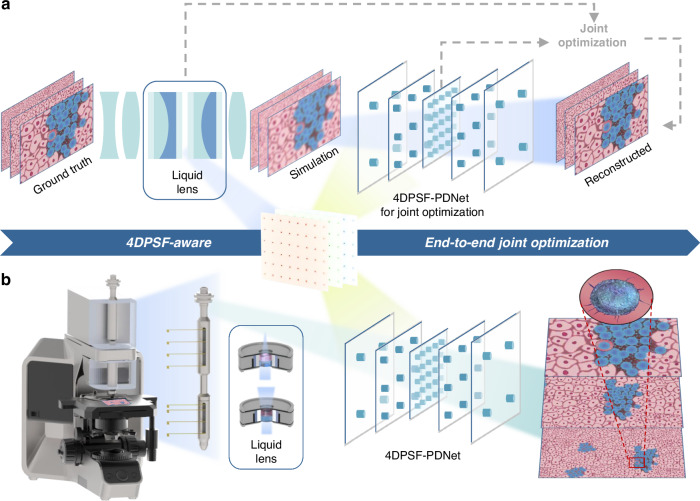
Schematic diagram of the adaptive continuous microscopy. **a** Pipeline of 4DPSF-aware end-to-end jointly optimization. **b** Concept map of the adaptive continuous zoom microscope

The proposed end-to-end joint optimization framework, guided by physical degradation information, as shown in Fig. 1a. The 4D point spread function obtained from optical simulations is used to guide the physical degradation-aware network for image reconstruction, enabling high-quality continuous imaging through joint optimization. A schematic of the proposed adaptive continuous zoom microscope is shown in Fig. 1b. The system is designed based on a liquid lens assembly that enables rapid image acquisition at multiple magnification levels through continuous optical zooming. In this work, the 4DPSF-PDNet is integrated to be the software module of the system to address the optical degradation occurring in different spatial regions at different magnifications. 4DPSF-PDNet takes the degraded image and the PSF corresponding to the spatial region as input, and performs adaptive noise suppression and aberration correction under varying zoom states and spatial conditions using the proposed physics-informed degradation-guided reconstruction framework. By training and optimizing the parameters of 4DPSF-PDNet, the system is capable of producing high-quality microscopic images across a continuous magnification range.

### Design of the continuous zoom microscope based on a liquid lens

The continuous optical zoom objective serves as the imaging unit of the proposed microscope and consists of an electrowetting liquid lens and a solid lens (Supplementary Fig. S1). The liquid lens functions as the core zoom element, responsible for the entire focal power variation of the system, while the solid lenses contribute partial optical power and provide higher-order aberration compensation.

According to the geometric configuration and the Young-Lippmann equation, the relationship between the focal length *f* of the electrowetting liquid lens and the applied voltage *U* can be expressed as:

1 $$f=\frac{-D({n}_{{\rm{c}}}-{n}_{{\rm{n}}})}{[\cos \,{\theta }_{0}+\varepsilon {\varepsilon }_{0}{U}^{2}/(2H\gamma )]}$$

where *n*_c_ and *n*_n_ represent the refractive indices of the conductive and non-conductive liquids, respectively. *θ*_0_ is the contact angle in the absence of an applied voltage, *ε*_*0*_ is the permittivity of free space, *ε* is the dielectric constant of the insulating layer, *H* is the thickness of the dielectric layer, *γ* denotes the interfacial tension between the two liquids, and *D* is the effective aperture of the electrowetting liquid lens. The focal length of the electrowetting lens is inversely proportional to the square of the applied voltage. Therefore, adaptive zooming can be achieved by adjusting the external voltage.

To achieve a larger zoom range while maintaining high resolution, a zoom imaging model with a movable relay image plane is proposed. This model is divided into a front zoom group and a rear zoom group, forming a two-degree-of-freedom zoom space. This structure enhances the system’s zoom flexibility and aberration compensation capability. The overall system magnification can be expressed as:

2 $$\beta {=(1-\Phi }_{f}\cdot u{)}^{-1}\times {[1+{\Phi }_{c}{({\Phi }_{f}+{u}^{-1})}^{-1}-{\Phi }_{f}\cdot d]}^{-1}$$

where Ф_*f*_ and Ф_*c*_ denote the optical powers of the front and rear zoom groups, respectively. *u* is the working distance of the system, and *d* is the distance between the centers of the front and rear zoom groups. During continuous optical zooming, the liquid lenses maintain a fixed object-image plane configuration, meaning both the working distance *u* and the center distance *d* remain constant. As a result, the system magnification is determined by the optical powers of the front and rear zoom groups.

The zoom ratio achievable under a fixed relay image plane is limited by the optical power variation range of the liquid lens. To further expand the zoom range, a movable relay image plane is introduced. When the relay image plane shifts toward the rear zoom group, the object distance of the front zoom group remains constant, while its image distance increases, thereby increasing its magnification. Simultaneously, the object distance of the rear zoom group decreases while its image distance remains unchanged, leading to an increase in its magnification as well. Conversely, when the relay image plane shifts in the opposite direction, the magnifications of both the front and rear zoom groups decrease. Therefore, by introducing a movable relay plane, the system achieves an extended range of magnification variation.

### Principle of the 4DPSF-PDNet

**Fig. 2:**
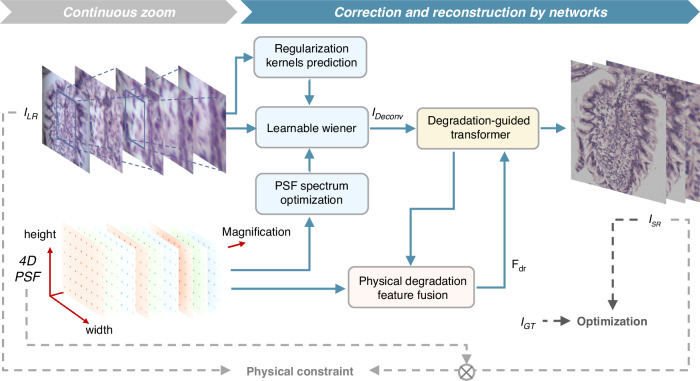
Structure and mechanism of the 4DPSF-PDNet

The structure and mechanism of the proposed 4DPSF-PDNet are shown in Fig. 2. The network integrates the advantages of physical priors and deep learning. We use the multi-wavelength, continuous-magnification, spatially-variant 4D PSF of the system as physical prior information, and then apply a Content-Adaptive, learnable Wiener filter in the frequency domain to preliminarily suppress noise and recover spatial structural information from continuously zoomed images. Based on the fusion of the PSF and features, degradation features that contain physical information are generated to further guide a multi-scale, multi-head self-attention mechanism for aberration correction and detail reconstruction.

### Content-adaptive learnable Wiener

Traditional deconvolution-based algorithms are very sensitive to the PSF and the noise in the image. PSFs are not entirely accurate due to factors such as manufacturing tolerances. Furthermore, they typically use a fixed, non-adaptive regularization kernel, which limits their stability and robustness when dealing with the wide variety of degradation characteristics that appear across the zoom range^38,39^. To address these issues, a data-driven, learnable Wiener filtering model, Content-adaptive learnable Wiener (CALW), is adopted to enable end-to-end trainable Wiener deconvolution. This model takes the PSF of the corresponding spatial region as input and performs optimization in the frequency domain. Together with a regularization convolution kernel that is adaptively generated based on image content, CALW performs channel-wise Wiener deconvolution. Channel-wise learnable parameters are introduced to further optimize the restoration process. This Wiener filter, which has a closed-form solution, is derived using variational methods and diagonalized in the Fourier domain as follows (Supplementary S2):

3 $$\hat{{\bf{x}}}={{\bf{F}}}^{H}(\frac{{{\bf{D}}}_{{\bf{K}}}^{\ast }{\bf{F}}{\bf{y}}}{{|{{\bf{D}}}_{{\bf{K}}}|}^{2}+{e}^{{\alpha }_{c}}{\sum }_{d=1}^{D}{|{{\bf{D}}}_{{{\bf{G}}}_{i}}|}^{2}})$$

where **F**∈ℂ^*N×N*^ and **F**^*H*^∈ℂ^*N×N*^ represent the Fourier transform matrix (DFT) and its inverse, respectively. Due to the presence of chromatic aberration, the PSFs for each channel exhibit discrepancies. To better accommodate the varying effects of chromatic aberration across different channels, CALW performs frequency-domain operations on both the degraded image and the PSF in a channel-wise manner. Independent learnable parameters *α*_*c*_ is assigned, and *c* = *r, g, b* for the red, green, and blue channels, respectively. Furthermore, both the optimization of the PSF and the estimation of the regularization kernel are incorporated into the learnable framework, which is implemented through two modules: PSF Spectrum Optimization and Regularization Kernels Prediction (Supplementary S3.1).

### Degradation-guided detail reconstruction

Convolutional neural network (CNN)-based deep learning methods for image restoration are limited in capturing long-range dependencies. Meanwhile, multi-head self-attention (MSA)-based approaches often suffer from insufficient detail handling and high computational overhead^45–47^. To address these limitations, a Degradation-guided Multi-Head Self-Attention mechanism (DGMSA) is proposed, and a Degradation-guided Transformer (DGT) is constructed. Additionally, Physical Degradation Feature Fusion (PDFF) is introduced, which performs frequency-domain fusion of PSF and features (Supplementary S3.2). This enables the generation of multi-scale degradation features incorporating physical degradation priors, which are used to guide the modeling of long-range correlations and interactions across spatial regions under varying degradation conditions.

Due to the global modeling capacity and enhanced adaptability, DGT can better accommodate complex and variable aberrations that arise across different spatial locations and magnification levels (Supplementary S3.3). By integrating degradation-aware priors derived from the PSF, the model achieves a more robust and generalizable aberration correction framework.

### Loss function

To ensure the physical plausibility of the generated content, a physics-constrained loss is defined, which penalizes discrepancies between the downsampled convolution of the super-resolution output image with the optimized PSF and the input degraded image. This constraint suppresses the generation of non-physical artifacts during training. To further preserve fine details and texture in the reconstructed images, the overall loss function combines the physics-constrained MSE loss with a multi-scale structural similarity loss and total variation loss. Together, these components form a combined loss function in the 4DPSF-PDNet (Supplementary S3.4).

### Performance test of the continuous zoom microscope

Starting from the variable relay image plane-based microscope imaging model, ray-tracing-based optimization with emerging deep optics optimization techniques to achieve physically guided optimal imaging performance is integrated. First, optical design is carried out using Zemax software. Following the conventional reverse ray-tracing method, the front and rear zoom groups are independently optimized. Each group integrates four liquid lenses, resulting in zoom ranges of 2× ~ 8× for the front group and 5× ~ 12.5× for the rear group. These two subsystems are then stitched using a dynamic pupil matching strategy. The stitching error is evaluated and compensated through inverse optimization, yielding a continuous optical zoom objective lens model covering a 10× ~ 100× range. Second, with the relay image plane set as a variable, pupil drift compensation is introduced, and a stitching-matching function is established to optimize the stitched system. This results in a set of candidate objective lens models with 10× ~ 100× continuous zoom capability. Third, the 4D PSFs of these candidate models are extracted and used as physical priors to inject into the 4DPSF-PDNet. Degraded simulation images exported from Zemax are further perturbed with noise and paired with the corresponding ground truth images to form training datasets. The candidate model with the best image quality is selected as the final optical design.

**Fig. 3:**
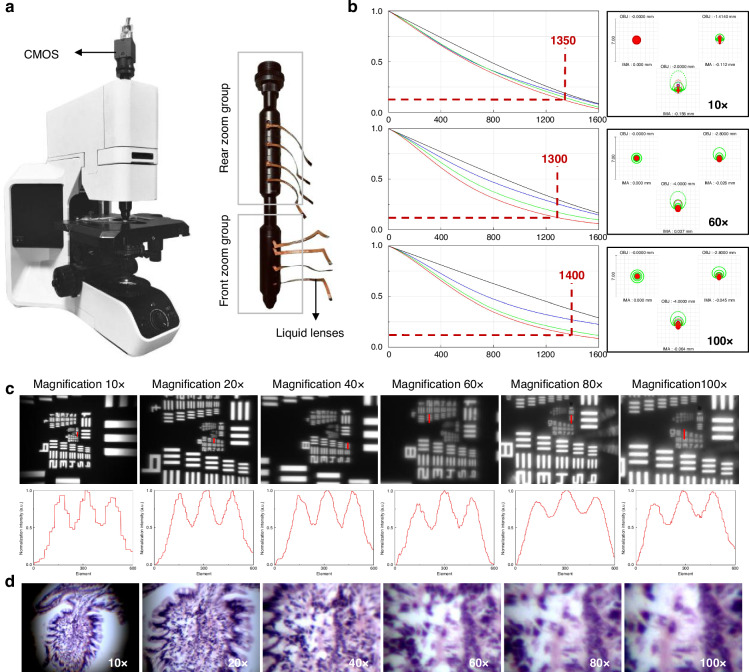
Fabrication and performance test of the continuous microscope. **a** Structure of the continuous optical zoom microscope and zoom objective lens. **b** MTF of the zoom objective lens at the magnifications of 10×, 60×, and 100×. **c** Resolution target image and analysis. **d** Imaging testing on small intestine slices

According to the design specifications, the optical model of the proposed microscope is simulated using Zemax, and the position variation curve of the relay image plane is obtained (Supplementary S4). Based on this model, the continuous optical zoom objective lens is fabricated. Taking mechanical constraints into account, the high-zoom-ratio continuous optical zoom microscope is integrated, as shown in Fig. 3a. The main structure of the microscope is constructed from aluminum alloy, which is equipped with a high-precision three-dimensional stage. The Abbe condenser provides a maximum numerical aperture (NA) of 1.1, and a bottom-mounted LED source offers transillumination across the 400 nm ~760 nm spectral range. The solid lenses are manufactured by DaHeng New Epoch Technology Inc. Both the front and rear zoom groups integrate four liquid lenses each, manufactured by Corning Inc., model Arctic-58N0, with an effective aperture of 5.8 mm and tunable optical power ranging from −5 D(m^-1^) to +10 D(m^-1^). The CMOS used in the experiments is the type of BFS-U3-32S4C-C produced by FLIR, CAN. The size of the CMOS is 1/1.8", and the pixel size is 3.45 μm. The highest resolution is 2048 pixels×1536 pixels.

System imaging quality evaluation can provide guidance for imaging experiments (Supplementary S5). The MTF (Modulation Transfer Function) curves and Spot Diagrams of the system are obtained under visible wavelengths (0.486, 0.587, and 0.656 μm) at magnification levels of 10×, 60×, and 100×, as shown in Fig. 3b, demonstrating that the designed system is capable of producing high-quality, diffraction-limited images.

In the first experiment, a resolution test target is used to evaluate system resolution. The resolution test pattern conforms to the MIL-STD-150A standard, set by USAF-1951. Based on the simulation results, a correspondence curve between the system magnification and the driving voltage of the liquid lens is obtained (Supplementary S6). We further fine-tune the driving voltages to achieve the final continuous optical zoom imaging results of the resolution test target across a magnification range of 10× ~ 100×, as shown in Fig. 3c. Moreover, the corresponding normalized intensity curve is obtained by using an algorithm. At lower magnifications, the imaging system offers a wide field of view, though minor distortion is observed at the field edges. The central field exhibited minimal aberrations and good image quality, resolving up to Element 1 of Group 9. The system resolution increases with magnification and reaches its maximum at 100×, resolving up to Element 3 of Group 10.

To further validate the performance of the proposed microscope, we conduct biological imaging experiments using the high-resolution, large zoom-ratio continuous optical zoom microscope. The images of small intestine slices are captured, as shown in Fig. 3d. During the continuous zoom process, the observed tissue consistently and clearly remains in the center of the field of view. In the high-magnification range (60× ~ 100×), the system aperture remains constant, leading to reduced light throughput and a noticeable decline in contrast, which results in a visually softer image. While a traditional solid lens can improve transmittance through optical coatings, a liquid lens generally has lower intrinsic transmittance, at approximately 90%, which can introduce significant ghosting effects. This is one of the key reasons for the observed contrast degradation at high magnifications. Nonlinear aberrations during the zooming process are another key factor leading to the decrease in the clarity of the image.

### Aberration correction results

**Fig. 4:**
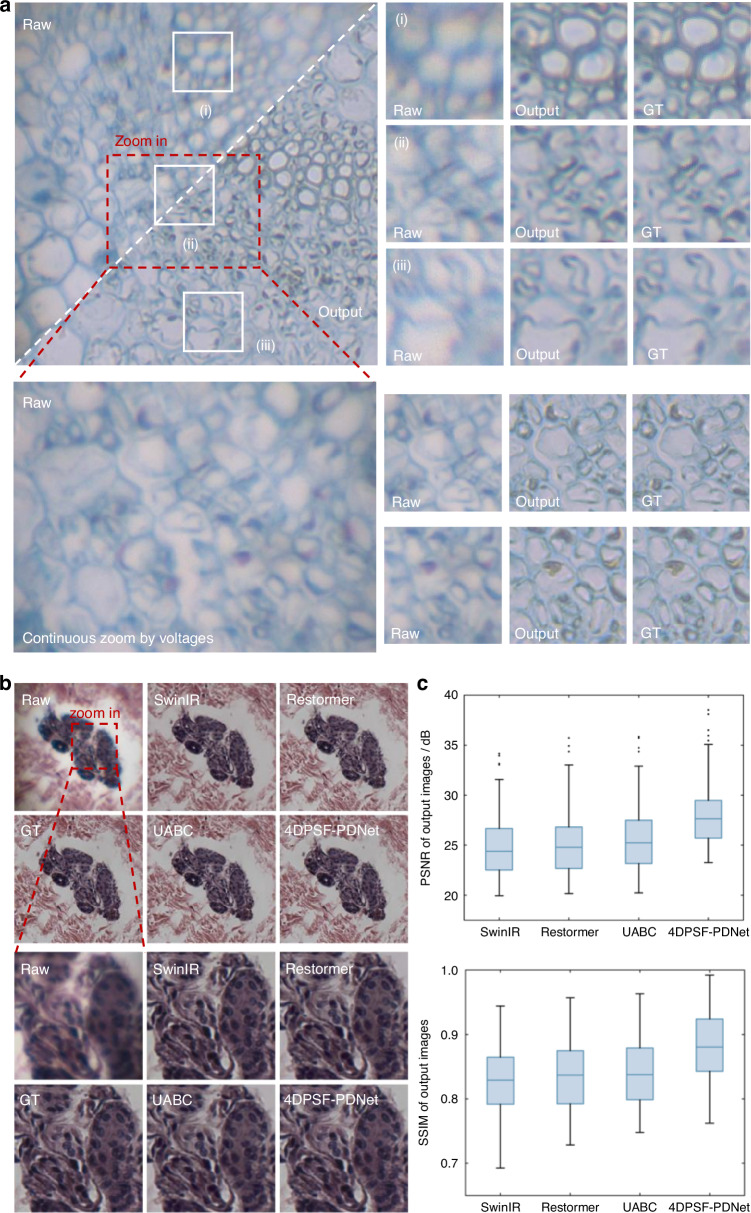
Network processing results. **a** Comparison of aberration correction and detail restoration at different magnifications during continuous zooming on a cross-section of a leaf sample. **b** Comparison of different state-of-the-art models on human skin sweat gland slice samples. **c** Quantitative comparison of different advanced models

The proposed 4DPSF-PDNet achieves effective aberration correction and detail restoration throughout the continuous zoom process. As shown in the Fig. 4a, at low magnifications, the model demonstrates strong suppression of longitudinal and axial chromatic aberrations, enabling accurate recovery of image information and supporting wide-field observation for initial sample assessment. At high magnifications, the model effectively restores fine image details, facilitating in-depth analysis of localized structures.

To validate the effectiveness of the proposed approach in simultaneously correcting aberrations and recovering fine textures, we compare 4DPSF-PDNet with state-of-the-art models. These include SwinIR^46^ and Restormer^47^, advanced image restoration models, and UABC^40^, which utilizes PSF to guide optical aberration correction. As shown in the Fig. 4b, under the influence of complex aberrations, the compared models struggled to accurately recover structural details, resulting in information loss, which affects the accuracy in microscopic observations. At high magnifications, since the models focus more on the restoration of fine structures, Restormer and UABC are less accurate in restoring colors. Although SwinIR maintains the correct color, it also fails to restore the degraded details. Under the guidance of the degradation prior, 4DPSF-PDNet is able to better perform adaptive aberration correction and detail restoration according to different degradation characteristics. Furthermore, we perform quantitative comparisons using Peak Signal-to-Noise Ratio (PSNR) and Structural Similarity Index (SSIM) (Supplementary S7.1). On the validation set comprising degraded images at multiple magnification levels, our model achieved a PSNR of 28.12 dB, which represents an improvement of approximately 2.5 dB over UABC (25.66 dB). In comparison with SwinIR and Restormer, the PSNR increase exceeds 3 dB, thereby demonstrating the superior performance of 4DPSF-PDNet. In addition, we conducted ablation studies to verify the effectiveness of the proposed CALW module (Supplementary S7.2).

## Discussion

In this work, an end-to-end joint optimization approach guided by physical priors is proposed, which greatly enhances both continuous zoom range and imaging quality to achieve an optimal combination of microscopic optics design and neural network. The resulting high dynamic range liquid lens–based continuous optical zoom microscope delivers rapid, high-quality imaging with a continuous zoom range of 10.6× ~ 101.4×. Together with the proposed 4DPSF-PDNet, the system adaptively corrects complex aberrations that vary with magnification and spatial location during continuous zooming. By incorporating the PSF into the network as a physical constraint, a physics-constrained loss function is constructed, which enables the network to more precisely correct complex dynamic aberrations while suppressing distortions and artifacts. 4DPSF-PDNet outperforms other image aberration correction models, demonstrating superior performance in both visual quality and quantitative metrics. Compared with existing state-of-the-art physics-guided image reconstruction methods, the proposed method achieves a 2.5 dB increase in PSNR.

Despite the effectiveness of our framework, several promising directions for future research remain. For instance, further exploration of richer degradation cues from the high-dimensional PSF representations could enhance performance. In addition, reducing the model’s inference time remains an important objective, which may require innovations in network architecture, such as incorporating lightweight modules or applying model compression techniques like knowledge distillation to reduce the number of parameters. It is also necessary to further develop deep learning-based methods to address the inherent optical problems of liquid lenses, such as complex aberrations and low contrast caused by low transmittance.

By combining physics-based priors with the strengths of deep learning, our approach leverages the PSF as a bridge to guide the joint optimization of optical design and neural network. In handling complex dynamic aberrations, the embedding of physical information into the correction network not only suppresses distortions and artifacts but also enables more accurate restoration. The proposed adaptive continuous microscope achieves both large-range continuous optical zooming and high-resolution imaging, demonstrating strong potential for applications in pathological diagnosis, cell analysis, and materials characterization.

## Materials and methods

### Training detail

We performed optical simulations based on Huygens’ principle using Zemax software, from which we extracted the system’s 4D PSF and established a 4D PSF dictionary for subsequent model training and testing. In the specific setup, the sampling interval of the image plane in the simulation was set to 3.45 μm, consistent with the image plane size of the FLIR BFS-U3-32S4C-C CMOS. The number of PSFs exported was determined by the sensor resolution (2048×1536), and we extracted PSFs corresponding to 256 × 256 patches. For each magnification level ranging from 10× to 100×, 48 PSFs (8×6) were computed for each different zoom state. Additionally, PSFs were extracted at three wavelengths (0.486, 0.588, and 0.656 μm) to represent degradation information across different spectral bands.

The model training described in this paper involved two distinct phases:

In the first phase, during end-to-end joint optimization, we used the Olympus BX53M microscope equipped with 50×/0.80NA and 100×/0.90NA objectives (in air) to capture approximately 120 ground truth (GT) images from 68 micro-samples (covering plant and animal specimens). Based on the acquired GT images, Zemax software simulated aberration images at different zoom states through ray tracing. Gaussian noise with standard deviations *σ* = 2−10 was randomly added to these images, followed by downsampling to obtain degraded images. Prior to training, data augmentation was applied to paired degraded input/GT images through horizontal/vertical flipping and rotation, producing approximately 12,000 degraded images of size 256 × 256 and their corresponding GT images of size 512 × 512 as paired training samples.

In the second phase of model training, we used the proposed microscope to capture images at magnifications ranging from 10× to 100× for 68 micro-samples. These images were paired with corresponding images captured by a commercial microscope to construct the training dataset. Considering that chromatic aberration and other optical distortions are more pronounced at lower magnifications, images captured at 50× magnification with the commercial microscope were used as GT for supervised training at low magnifications (10×–50×). For high magnifications (50×–100×), where detail recovery is emphasized, images captured at 100× magnification with the commercial microscope served as the GT for supervised training. This strategy allowed the model to generalize better across different magnifications and field-of-view ranges. In line with the first phase, after downsampling the images captured with the proposed microscope, data augmentation was applied to the paired input/GT images through horizontal/vertical flipping and rotation. This resulted in approximately 12,000 degraded images of size 256 × 256 and their corresponding GT images of size 512 × 512 as paired training samples.

During model training, for each image patch, we retrieved the corresponding PSF from the established 4D-PSF dictionary and used it as the model input corresponding to the degraded input image. The two training phases used consistent training settings and testing procedures, and used graphics processing units (GPUs) to accelerate the training and testing processes through CUDA. All networks were implemented in Python with the PyTorch framework. Training was performed on four NVIDIA Tesla V100 (16GB memory), with a batch size of 8, and the model was trained for 200 epochs. The Adam optimizer (*β*_1_ = 0.9, *β*_2_ = 0.999) was used for optimization, with an initial learning rate of 2 × 10^−5^, adjusted using a cosine annealing strategy that restarted every 20 epochs. During evaluation, we tested 16 sample images of size 512 × 512 at six magnifications (10×, 20×, 40×, 60×, 80×, and 100×) and evaluated performance using PSNR and SSIM metrics.

## Supplementary information

Supplementary for Large zoom ratio and adaptive aberration correction microscope using 4DPSF-aware Physical Degradation-guided Network

## Data Availability

The data that support the findings of this study are available from the corresponding author upon reasonable request.
