# Supplementary material for: Large zoom ratio and adaptive aberration correction microscope using 4DPSF-aware Physical Degradation-guided Network
Source: Light Sci Appl. 2026 Mar 3;15:140. doi: 10.1038/s41377-025-02155-8 (PMC12953653; doi:10.1038/s41377-025-02155-8)
Supplement: Supplementary file 1 — Supplementary for Large zoom ratio and adaptive aberration correction microscope using 4DPSF-aware Physical Degradation-guided Network [file 41377_2025_2155_MOESM1_ESM.docx]

SUPPLEMENTARY INFORMATION

**Large zoom ratio and adaptive aberration correction microscope using 4DPSF-aware Physical Degradation-guided Network**

Dong-Xu Yu^1,†^, Zhao Jiang^2,†^, Yi Zheng^1^, Hao-Ran Zhang^1^, Rong-Qiang Li^1^, You-Ran Zhao^1^, Xiao-Ke Lu^1^, Yu-Cheng Lin^1^,

Chao Liu^1,*^, and Qiong-Hua Wang^1,*^

*^1^ School of Instrumentation and Optoelectronic Engineering, Beihang University, Beijing 100191, China.*

*^2^ School of Remote Sensing Science and Technology, Aerospace Information Technology University, Jinan 250299, China*

^†^*These authors contributed equally to this work.*

**Correspondence: C Liu, E-mail: chaoliu@buaa.edu.cn;*

*QH Wang, E-mail: qionghua@buaa.edu.cn*

19 pages, 12 figures S1-S12, 3 tables S1-S3

**S1:** **Schematic of the continuous zoom microscope based on liquid lens**

The optical structure of the continuous zoom microscope based on liquid lens is shown in Fig. S1. The core component of the microscope is a zoom objective, which integrates seven single-element glass lens, four doublet lens, and eight electrowetting liquid lens. Each liquid lens consists of a conductive liquid and a non-conductive liquid, and is actuated through the electrowetting effect. The inner cavity of the liquid lens is coated with a dielectric layer and a hydrophobic layer. The dielectric layer prevents electrical breakdown under external voltage, while the hydrophobic layer ensures a large initial contact angle for the conductive liquid. The contact angle between the conductive liquid and the cavity can be modulated by an external voltage. The change in contact angle leads to a variation in the curvature of the liquid–liquid interface. According to the Young–Lippmann equation, the relationship between the contact angle *θ*_1_ and the applied voltage *U* can be described as follows:

$$\cos\theta_{1}=\cos\theta_{0}+\frac{U^{2}\varepsilon\varepsilon_{0}}{2H\gamma_{12}}$$

where *θ*_0_ is the initial contact angle without applied voltage, *ε* and *ε*_0_ are the dielectric constants of the insulating layer and free space, respectively, *H* is the thickness of the dielectric layer, and *γ*_12_ is the interfacial tension between the conductive and non-conductive liquids.

Based on the geometrical relationships and the Young–Lippmann equation, the relationship between the focal length *f* of the electrowetting liquid lens and the applied voltage *U* can be calculated as:

$$f=\frac{-D\left(n_{c}-n_{n}\right)}{\left[\cos\theta_{0}+\frac{\varepsilon\varepsilon_{0}U^{2}}{2H\gamma}\right]}$$

where *D* represents the effective aperture of the electrowetting liquid lens, *n_c_* and *n_n_* are the refractive indices of the conductive liquid and the non-conductive liquid, respectively. It can be observed that the focal length of the electrowetting lens is inversely proportional to the square of the applied voltage. By adjusting the external voltage, adaptive zooming can be achieved. However, the optical power tuning range of a single liquid lens is limited. Therefore, in this work, continuous optical zoom microscopy is realized without any mechanical movement by jointly controlling multiple liquid lens through coordinated voltage modulation.


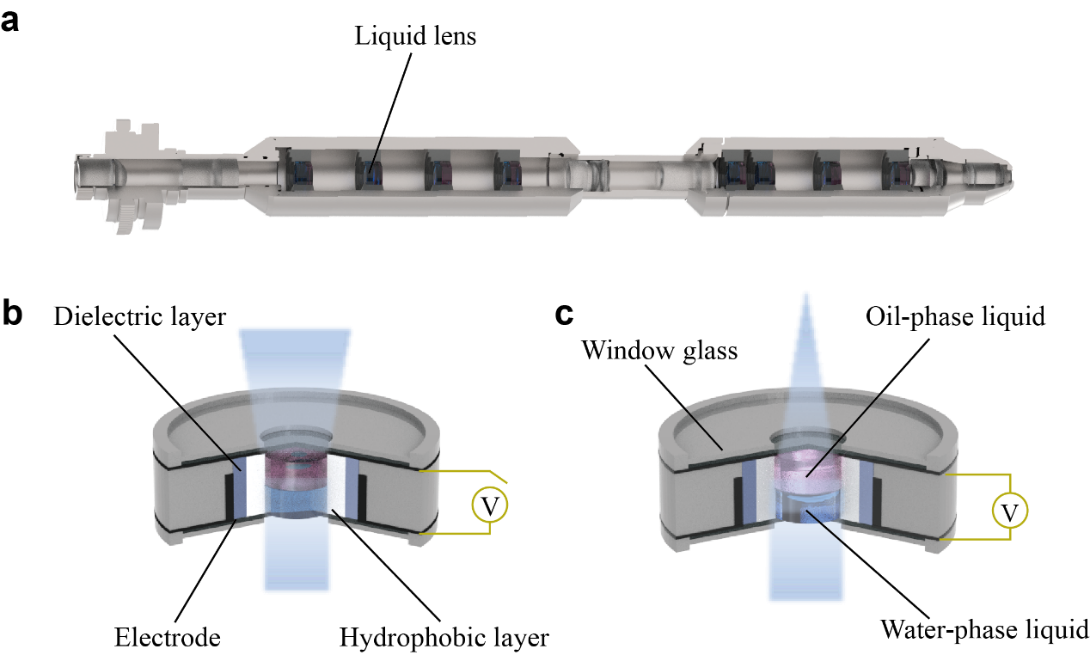


**Fig. S1** **Optical structure of the adaptive microscope. a** Structure of the liquid microscope. **b** Liquid lens working in concave lens state. **c** Liquid lens working in convex lens state.

**S2: Deduction of Wiener deconvolution using variational method**

During the imaging degradation process, in addition to aberrations such as blur and chromatic distortion caused by the PSF, the image quality is further degraded by noise introduced by the CMOS sensor. This degradation process can be represented as the following equation:

$$\mathbf{y=kx+n}$$

where ​**y**∈ℝ*^N^* denotes the degraded observed image, **k**∈ℝ*^N×N^* represents the point spread function (PSF) matrix, **x**∈ℝ*^N^* ​is the ideal high-resolution image to be recovered, and n~(0, *σ*^2^) denotes the noise, which is assumed to follow a Gaussian distribution. For the sake of mathematical derivations, we assume that **x** and **y** have been raster scanned using a lexicographical order, and they correspond to vectors of *N* dimensions.

Deconvolution is an ill-posed inverse problem, which in this work is addressed using a variational approach. The core idea of variational methods is to find the minimum or maximum of a functional by analyzing its infinitesimal variations, known as variational derivatives. In our approach, the solution is obtained by minimizing a well-defined objective functional:

$$\hat{\mathbf{x}}=\arg\min_{\mathbf{x}} \frac{1}{2}\left\|\mathbf{y-Kx}\right\|_{2}^{2}+\lambda r\left(x\right)$$

The first term corresponds to the **data fidelity term**, which measures the closeness between the solution and the observed data. The second term represents the **regularizer**, which models any prior knowledge about the ground truth image. The parameter *λ* is a **trade-off coefficient** that determines the contribution of the regularization term to the overall solution estimation. Here, we aim to integrate prior information learned directly from available training data in a supervised manner with deep learning strategies.

We explicitly define the penalty function as the squared L2-norm, thereby yielding a Tikhonov regularizer:

$\hat{\mathbf{x}}=\arg\min_{\mathbf{x}} \frac{1}{2}\left\|\mathbf{y-Kx}\right\|_{2}^{2}+\lambda\sum_{d=1}^{D} \left\|\mathbf{G}_{d}\mathbf{x}\right\|_{2}^{2}$,

where **G***_d_*∈ℝ*^N×N^* is the convolution matrix corresponding to the learnable convolution kernel. The minimization problem above admits a closed-form solution corresponding to the Wiener–Kolmogorov deconvolution filter:

$$\hat{\mathbf{x}}=\left(\mathbf{K}^{T}\mathbf{K}+\lambda\sum_{d=1}^{D} \mathbf{G}_{d}^{T}\mathbf{G}_{d}\right)^{-1}\mathbf{K}^{T}\mathbf{y}$$

where **K**^T^ and **G**T *d* denote the adjoint matrices of **K** and **G***_d_*, respectively. Solving Equation (S6) requires inverting large matrices, which can be computationally expensive. To address this, we reformulate the closed-form solution in the Fourier domain, making the computation of the Wiener filter fast and efficient, with low computational complexity for signal recovery. Assuming periodic boundary conditions for the image, both **K** and **G***_d_* can be treated as circulant matrices, which can be diagonalized in the Fourier domain as follows:

$$\mathbf{K}=\mathbf{F}^{H}\mathbf{D}_{\mathbf{K}}\mathbf{F},\mathbf{D}_{\mathbf{K}}=\mathbf{FS}_{\mathbf{K}}\mathbf{P}_{\mathbf{K}}\mathbf{k};\mathbf{G}_{d}=\mathbf{F}^{H}\mathbf{D}_{\mathbf{G}_{d}}\mathbf{F},\mathbf{D}_{\mathbf{G}_{d}}=\mathbf{FS}_{\mathbf{G}_{d}}\mathbf{P}_{\mathbf{G}_{d}}\mathbf{g}_{d}$$

where **F**∈ℂ*^N×N^* is the Fourier (DFT) matrix, and **F***^H^*∈ℂ*^N×N^* is its inverse. **D_k_**∈ℂ*^N×N^* and **D_G_***_d_*∈ℂ*^N×N^* are diagonal matrices, and **S_k_**∈ℝ*^N×N^*, **S_G_***_d_*∈ℝ*^N×N^* are the corresponding circulant shift operators. **P_k_**∈ℝ*^N×M^* and ​​**P_G_***_d_*∈ℝ*^N×Ld^* denote the zero-padding operators, **k**∈ℝ*^M^* is the blur kernel, and **g***_d_*∈ℝ*^M^* is the regularization convolution kernel. Similarly, the trade-off coefficient $\lambda=e^{\alpha_{c}}$ is treated a learnable parameters for three channels, constrained to be positive to ensure stability during training. Based on the derivation above, we obtain the following formula:

$$\hat{\mathbf{x}}=\mathbf{F}^{H}(\frac{\mathbf{D}_{\mathbf{K}}^{*}\mathbf{Fy}}{\left|\mathbf{D}_{\mathbf{K}}\right|^{2}+e^{\alpha_{c}}\sum_{d=1}^{D} \left|\mathbf{D}_{\mathbf{G}_{i}}\right|^{2}})$$

where **D* k** denoting the Hermitian transpose of the **D_k_**, and division is performed element-wise.

**S3: Details of the 4DPSF-PDNet**

**S3.1: Additional description of the Content-Adaptive Learnable Wiener Deconvolution**

Traditional Wiener deconvolution methods are highly sensitive to noise parameters and the input PSF. In practice, noise parameters are often difficult to estimate accurately, and the simulated PSF may contain modeling errors, which can lead to instability in the deconvolution process and degraded restoration performance. One major limitation of conventional Wiener filtering lies in its fixed design of the regularization kernel, where a uniform analytical form is applied to all images regardless of content variations. This lack of adaptability makes it difficult to optimize the restoration process for images with different degradation characteristics, thereby limiting both stability and robustness^1^.


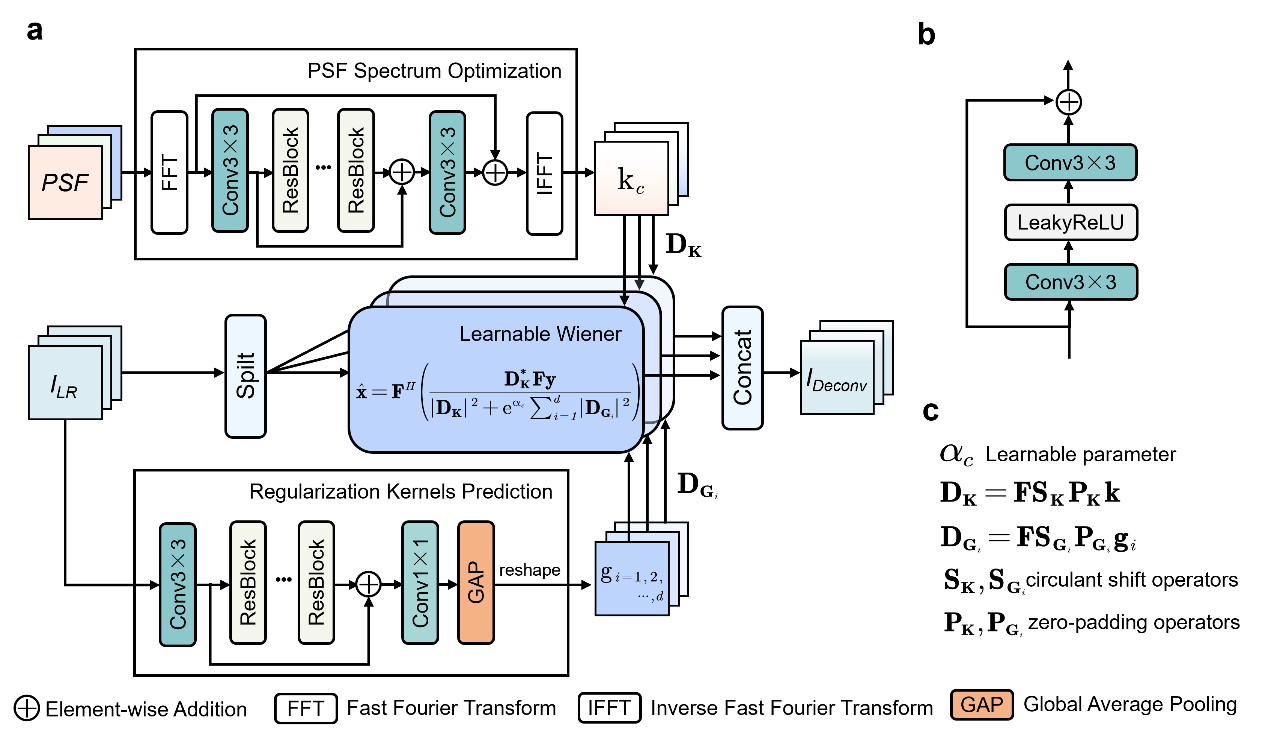


**Fig. S2 Detailed schematic structures of the Content-Adaptive Learnable Wiener Deconvolution. a** Structure of the PSF spectrum optimization, Regularization kernels prediction and learnable wiener. **b** Structure of the ResBlock. **c** Explanation of relevant parameters.

To address these issues, we incorporate both Regularization Kernels Prediction and PSF Spectrum Optimization into the learnable framework, as shown in Fig. S2. In Regularization Kernels Prediction, a conv3×3 is first applied to the input image to extract local spatial features. Then, deep feature representations are refined using a residual block (ResBlock) with skip connections. A conv1×1 is subsequently used to compress and integrate channel-wise information, yielding compact and discriminative feature representations. These features are globally aggregated using Global Average Pooling (GAP) to produce vectorized representations, which are then reshaped into content-adaptive regularization kernels with dimensions 𝑑×𝑚×𝑚. In our implementation, we set the number of filters to 𝑑=8 and the kernel size to 𝑚=3.

In PSF Spectrum Optimization, the PSF is optimized in the frequency domain, as Wiener deconvolution is inherently formulated in the frequency space and its core equations rely on the frequency response of the PSF. By optimizing the PSF spectrum directly, the network can better adjust to key frequency components. Moreover, in the frequency domain, the relationship among the PSF, noise, and system response is more explicit, facilitating the learning and enhancement of critical frequency features. A residual connection strategy is employed to ensure that the optimized PSF retains its physical consistency while improving reconstruction performance during the deconvolution process.

**S3.2: Description of the Physical Degradation Feature Fusion**

The structure of Physical Degradation Feature Fusion (PDFF) is shown in Fig. S3a, both the PSF and the input features are transformed into the frequency domain, where they are concatenated channel-wise. Convolutional and residual operations (ResBlocks) are then used to enable deep feature fusion between the two, allowing the network to adaptively rescale each feature map under PSF guidance based on the combined contribution of all frequency components in the power spectrum. The low transmittance of liquid lens leads to reduced light throughput, which in turn lowers imaging brightness, decreases contrast, and potentially causes stray light that can form ghost images, negatively impacting imaging quality. Liquid lens also exhibits dynamic and complex aberration types under different driving voltages, which is an inherent optical defect in their imaging system. At each feature scale, the model adaptively rescales each feature map based on the comprehensive contribution of all frequency components in the power spectrum. This process can suppress low-frequency ghost information, thereby highlighting effective image details. In contrast, conventional spatial-channel attention (SCA) mechanisms calculate rescaling factors only from the average intensity of feature maps, which corresponds to the zero-frequency (DC) component^2^.


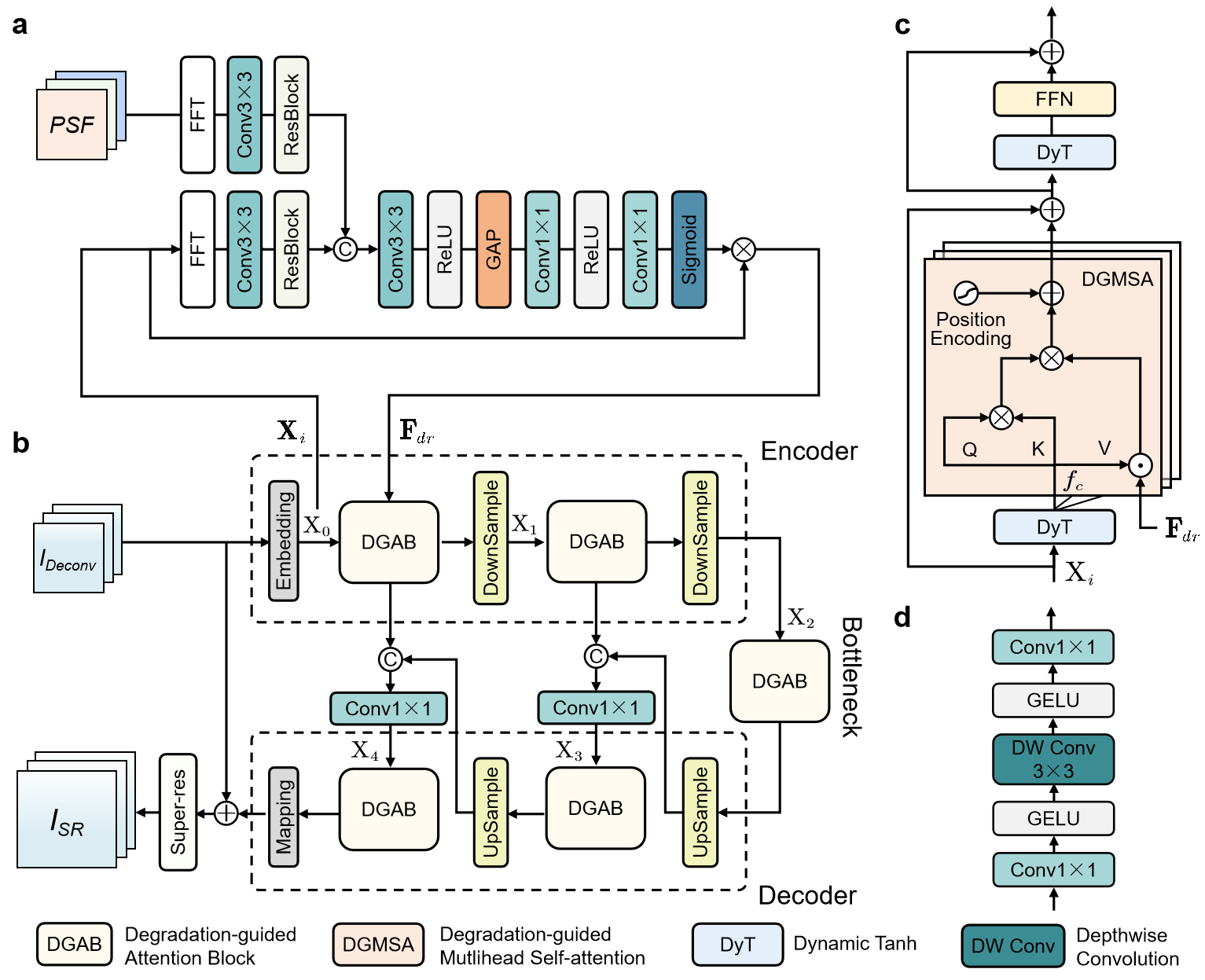


**Fig. S3 Detailed schematic structures of the Degradation-guided Detail Reconstruction.** a Structure of the Physical Degradation Feature Fusion. b Structure of the degradation-guided transformer. c Structure of the degradation-guided attention block. d Structure of the feedforward neural network.

In PDFF, the PSF and the deconvolved image are first transformed from the spatial domain to the frequency domain using the Fast Fourier Transform (FFT). This facilitates the capture of the PSF’s energy distribution characteristics in the spectral domain and enables a better assessment of the deconvolution results across both high- and low-frequency bands. A conv3×3 is then applied to each frequency-domain feature map to extract local spatial–frequency mixed information. Next, the two feature streams are passed through separate ResBlocks, where multiple conv layers and activation functions, combined with skip connections, allow for deep refinement of the frequency-domain features. This approach preserves key spectral components while suppressing irrelevant or noisy signals. The refined feature maps are then concatenated along the channel dimension, producing a joint frequency-domain representation that encodes physical degradation information. This fused feature map is further processed using a conv3×3 and ReLU activation to achieve channel-wise integration. GAP is then applied to aggregate global information, forming a compact and discriminative vector representation. A conv1×1 is used to reduce the number of channels, followed by a ReLU activation and another conv1×1 to restore the original channel size. Finally, a Sigmoid activation function is applied to generate a normalized channel attention weight matrix. This matrix is then multiplied, channel by channel, with the input features in the original spatial domain to achieve adaptive enhancement or suppression across channels, resulting in the final degradation-aware feature representation.

**S3.3: Additional Description of the Degradation-guided Transformer**

The Degradation-guided Transformer (DGT) adopts a U-Net style encoder–decoder architecture to perform aberration correction and super-resolution detail reconstruction through multi-scale feature extraction and reconstruction, as shown in Fig. S3b. The model takes as input the RGB image obtained from the first-stage deconvolution process and maps it into a high-dimensional feature space via a convolutional embedding layer. This is followed by a cascade of encoder, bottleneck, and decoder modules. To avoid information loss during downsampling, skip connections are used between the encoder and decoder. Additionally, a long skip connection is applied at the output stage to perform residual addition between the input image and the output features. Finally, the upsampling module is used to obtain output image with high resolution.

The basic unit of DGT is the Degradation-guided Attention Block (DGAB), which consists of Dynamic Tanh (DyT) layer, Degradation-guided Multi-Head Self-Attention mechanism (DGMSA), DyT and feed-forward network (FFN). The structure of FFN is shown in Fig. S3d. Residual connections are applied after both the DGMSA and the FFN to facilitate stable gradient flow. In this architecture, the pre-normalization operation is performed using the DyT layer instead of conventional normalization layers. According to recent research, the DyT layer not only reduces computational complexity but also improves model performance^3^. The DGMSA is responsible for realizing the degradation-guided multi-head self-attention mechanism.

As shown in Fig. S3C, the obtained degradation features **F***_dr_*∈*^H×W×C^* ​are reshaped into tokens **X**∈*^HW×C^*, which are then split and injected into *k* attention heads:

$$\mathbf{X}=\left[\mathbf{X}_{1},\mathbf{X}_{2},\cdots,\mathbf{X}_{k}\right]$$

where ，$d_{k}=\frac{C}{k}$ and $i=1,2,\cdots,k$. For features at different spatial resolutions, a conv 4×4 layer with stride = 2 is applied to downsample **F***_dr_*, ensuring spatial size alignment. Due to the nontrivial computational cost of global multi-head self-attention (MSA), the application of Transformer-based models in image restoration has been limited. To address this issue, we treat each single-channel feature map as a token and compute self-attention accordingly. Then For each head *i*, three fully connected layers *f_c_* without bias are used to linearly project **X***_i_* into query elements , key elements , and value elements  as

$\mathbf{Q}_{i}=\mathbf{X}_{i}\mathbf{W}_{\mathbf{Q}_{i}}^{T}$, $\mathbf{K}_{i}=\mathbf{X}_{i}\mathbf{W}_{\mathbf{K}_{i}}^{T}$,

where represent the learnable parameters of the fully connected layers and T denotes the matrix transpose.

We observe that different regions of the same image may be affected by different types of degradation. Regions with less severe degradation can provide semantic context to enhance regions suffering from more significant degradation. Therefore, we use the degradation feature **F***_dr_* to encode degradation information and guide the computation of self-attention by modeling the interactions among regions with varying degradation conditions.

To align with the shape of **X**, we reshape **F***_dr_* into **Y**∈ℝ*^HW×C^*, and then split it into *k* attention heads:

$$\mathbf{Y}=\left[\mathbf{Y}_{1},\mathbf{Y}_{2},\cdots,\mathbf{Y}_{k}\right]$$

where , $i=1,2,\cdots,k$. Then the self-attention for each head *k* is formulated as:

$$Attention\left(\mathbf{Q}_{i},\mathbf{K}_{i},\mathbf{V}_{i},\mathbf{Y}_{i}\right)=\left(\mathbf{Y}_{i}\odot\mathbf{V}_{i}\right)softmax\left(\frac{\mathbf{K}_{i}^{T}\mathbf{Q}_{i}}{\alpha_{i}}\right)$$

where *α_i_*∈ℝ^1^ is a learnable parameter that adaptively scales the matrix multiplication. Subsequently, *k* heads are concatenated to pass through an *f_c_* layer and then plus a positional encoding **P**∈ℝ*^HW×C^* (learnable parameters) to produce the output tokens **X**_out_∈ℝ*^HW×C^*. Finally, we reshape **X**_out_ to derive the output feature **F**_out_∈ℝ*^H×W×C^*.

The encoder of DGT adopts a stage-wise structure. In each stage, features are first enhanced using a DGAB module. This is followed by a strided conv4×4 layer for downsampling, during which the auxiliary degradation features are also downsampled in parallel. As the resolution is halved stage by stage, the number of channels is doubled, enabling the extraction of both rich local and global contextual information. The decoder is designed symmetrically to the encoder. Each decoding stage begins with an upsampling operation via a strided deconv2×2 layer, followed by a conv1×1 layer for feature fusion. Skip connections are used to merge features from the corresponding encoder stage into the current decoder stage, compensating for any information loss that may have occurred during downsampling. The fused features are then refined by DGAB to progressively restore spatial resolution and fine image details. The bottleneck module, composed of DGAB, further integrates global information and enhances the network’s ability to model detailed structures. It provides the deepest semantic features for the entire network.

In the output mapping stage, a conv3×3 is applied to project the high-dimensional features from the decoder back to the desired number of output channels. The result is then combined with the input image through residual addition. A dedicated upsampling module comprising conv3×3, PixelShuffle, and LeakyReLU activation is then applied to perform 2× super-resolution reconstruction, producing the final high-quality restored image. Benefiting from multi-scale contextual information extracted from the input and guided by the PSF, our model is able to better interpret the varying types of degradations introduced by the PSF in different spatial regions, and effectively handle more complex degradation recovery and image reconstruction tasks.

**S3.4: Description of the model inference complexity**

The main computational complexity of the model comes from the DGMSA in the proposed DGT, and its computational complexity is mainly the k times of multiplication of the two matrices in formula S12, that is, and . Its computational complexity can be expressed as:

the computational complexity of global MSA (G-MSA) can be expressed as follows:

from the comparison of S13 and S14, we can find that the computational complexity of DGMSA is linearly related to *HW*, while G-MSA is linearly related to (*HW*)^2^, which will bring more computational complexity. Our DGMSA can simplify the calculation process and thus shorten the inference speed.

Meanwhile, the normalization operation in the model is also computationally intensive. In our model, we use DyT to replace the normalization used in the traditional Transformer model. DyT is an element-by-element operation formula as follows:

$$\mathrm{DyT}\text{（}x\text{）}=\text{ }\gamma*\tanh\text{（}\alpha x\text{）+}\beta$$

where α is a learnable scalar parameter, γ and β are learnable, per-channel vector parameters. It does not require the calculation of the mean and variance of the input data. Traditional normalization methods (such as layer normalization) typically require the calculation and storage of the mean and variance of the entire batch, which increases memory usage and data transmission bandwidth consumption, especially when dealing with large batches and deep networks such as Transformers. DyT, on the other hand, does not require the calculation of the mean and variance of the input data and only requires the storage of a small number of learnable parameters, resulting in lower memory usage and more efficient data transmission. This not only speeds up computation during model training and inference, but also reduces the memory bandwidth bottleneck during model inference.

**S3.5: Description of the loss functions of the 4DPSF-PDNet**

To better preserve image details and texture, in addition to the mean squared error (MSE) loss, we incorporate multi-scale structural similarity (MS-SSIM) loss and total variation (TV) loss into our overall objective. Together with the physics-constrained loss, these components form a composite loss function that jointly promotes visual quality and physical consistency.

The MSE loss is a commonly used metric in deep learning-based image generation tasks, which optimizes the peak signal-to-noise ratio (PSNR) by calculating the difference in pixel values between the generated image and the target image. However, although the PSNR of the generated image can be optimized to a higher value, it often introduces over-smoothing effects, resulting in the loss of fine textures and structural details. To address this limitation, we introduce MS-SSIM loss to better preserve structural consistency across multiple scales. This metric evaluates similarities in luminance, contrast, and structure, aligning better with human perceptual judgment of image quality. Unlike pixel-level losses, MS-SSIM emphasizes the preservation of global structure and texture. Its multi-scale formulation further enables accurate assessment of structure at various resolutions. In addition, TV loss serves as a smoothing regularizer to suppress high-frequency noise and visual artifacts, thereby enhancing the perceptual quality of the reconstructed image. To enforce physical consistency, the physics-constrained loss is formulated based on PSF priors. By convolving the output image with the PSF and downsampling the result to compare it with input image via MSE, we effectively penalize unrealistic details and hallucinated structures during training. The loss function of the 4DPSF-PDNet is constructed by combining the MSE loss, the MS-SSIM loss, the TV loss, and the physics-constrained loss, constitute the combined loss used to train the network.

In 4DPSF-PDNet, the MS-SSIM loss is employed to evaluate the similarity between the predicted and target images across multiple scales in terms of structure, contrast, and luminance. Compared to simple pixel-level losses, MS-SSIM better preserves global structure and fine texture details. Moreover, its multi-scale formulation allows the network to effectively capture structural features at different resolutions. The formulation of the MS-SSIM loss is as follows:

$$Loss_{\mathrm{MS}}=1-\prod_{m=1}^{M} (\frac{2\mu_{\text{g}}\mu_{\text{t}}+c_{1}}{\mu_{\text{g}}^{2}+\mu_{\text{t}}^{2}+c_{1}})^{\beta_{\text{m}}}(\frac{2\sigma_{\text{gt}}+c_{2}}{\sigma_{\text{g}}^{2}+\sigma_{\text{t}}^{2}+c_{2}})^{\gamma_{\text{m}}}$$

where *m* is a scaling factor, and the height and width of the input image are scaled by a factor of 2^m-1^ (*m*=1, ..., *M*), *μ*_g_ and *μ*_t_ represent the mean values of the generated and the target image, respectively, *σ*_g_ and *σ*_t_ represent the standard deviations of the generated and the target image, respectively, *σ*_gt_ represents the covariance of the generated and the target image, *c*_1_ and *c*_2_ are two constant terms that are used to prevent the denominator from being zero, *β*_m_ and *γ*_m_ stand for the relative importance of the mean and variance terms.

The TV loss makes the generated image visually closer to the target image. The basic idea of the TV loss is to utilize the total variance of the image as a measure of the smoothness of the image, thus suppressing noise and artifacts. In the 4DPSF-PDNet, the TV loss is used as the regularizer. The TV loss is expressed as follows:

$$Loss_{\text{TV}}=\sum_{u,v} \sqrt{(p_{\text{u,v-1}}-p_{\text{u,v}})^{2}+(p_{\text{u+1,v}}-p_{\text{u,v}})^{2}}$$

where *p*_u,v_ represents a pixel point of the input image. The TV loss calculates the square root of the sum of the squares of the differences between each pixel point *p*_u,v_ and the neighboring pixels *p*_u,v-1_ in the horizontal direction and *p*_u+1,v_ in the vertical direction, respectively. The TV loss is obtained by summing up all the pixel points after completing the calculation.

To ensure the physical plausibility of the generated content and the correctness of sample observation, we define a physics-constrained loss. This loss is computed by downsampled convolving the output super-resolution image with the optimized PSF, and then calculating the mean squared error with the corresponding input degraded image​. This constraint effectively prevents the generation of non-physical artifacts during training. The physics-constrained loss is expressed as follows:

$$Loss_{PC}=\left\|I_{DR}-\left(I_{HR}\otimes PSF_{opt}\right)\downarrow\right\|_{2}^{2}$$

where *PSF*_opt_ refers to the optimized PSF obtained through PSF Spectrum Optimization corresponding to the spatial region, ↓ refers to the downsampling operation, *I*_HR_ denotes the high-resolution image predicted by the model, and *I*_DR_ represents the degraded input image.

In summary, the loss function of the 4DPSF-PDNet can be expressed as follows:

$$Loss=\lambda_{1}Loss_{MSE}+\lambda_{2}Loss_{MS}+\lambda_{3}Loss_{TV}+\lambda_{4}Loss_{PC}$$

where *λ*_1_, *λ*_2_, *λ*_3_ and *λ*_4_ are the coefficients of the MSE loss, the MS-SSIM loss, the TV loss and the physics-constrained loss, respectively. In the 4DPSF-PDNet, *λ*_1_, *λ*_2_, *λ*_3_ and *λ*_4_ are set to 1, 0.5, 1×10^-5^ and 0.1, respectively.

Furthermore, given that the 4DPSF-PDNet is trained using pairs of degraded (low-contrast) images and high-quality, high-contrast ground truth images. To minimize the loss function during training, the 4DPSF-PDNet must learn a mapping that not only corrects for aberrations but also restores the fine details and spatial structures present in the ground truth. This process of structure and detail restoration is fundamentally a form of contrast enhancement.

**S4: Optical simulation of the continuous zoom microscope based on liquid lens**

According to the zoom microscopy imaging model, the optical power is appropriately distributed to complete the independent design of the front and rear zoom groups. After optimizing the stitching process using the stitching-matching function, an initial structure of the zoom microscope objective is obtained. A new evaluation function is then constructed to jointly optimize the position of the relay image plane and the curvature radii of the liquid–liquid interfaces of the eight liquid lens. This process yields optimized simulation results for different magnification levels. The detailed 3D layout is shown in Fig. S4.


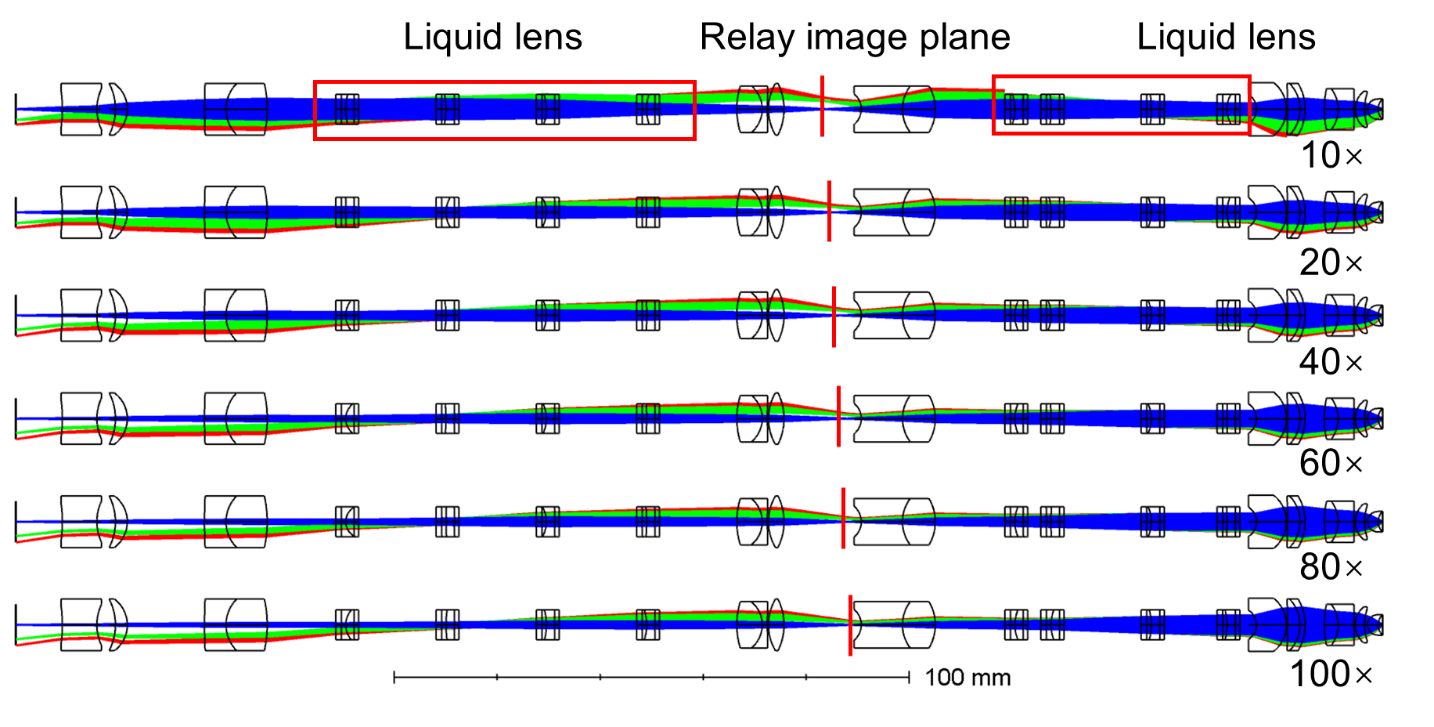


**Fig. S4 Simulation results of the zoom objective lens at the magnifications of 10×, 20×, 40×, 60×, 80×, and 100×.**

Construct a cooperative optimization operand for the front zoom group and the rear zoom group to ensure that the exit Angle of the front diploid is consistent with the incident Angle of the rear zoom group. Meanwhile, it is necessary to ensure that the incident pupil of the front zoom group and the exit pupil of the rear zoom group are at the same position, and the aperture Angle of the exit light of the front zoom group is smaller than that of the incident light of the rear zoom group. The positional variation relationship of the relay image plane is reflected in the lengths of the front and rear zoom groups, as shown in Fig. S4.

**
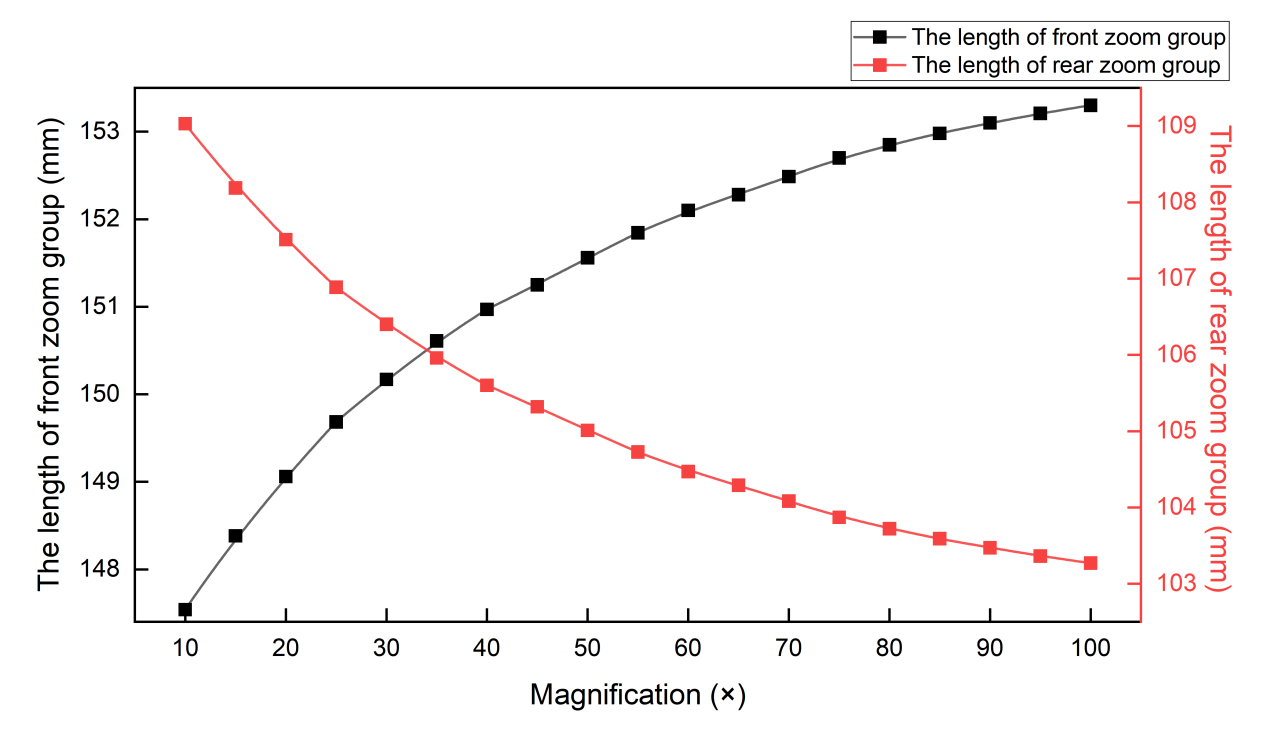
**

**Fig. S5 The relationship between magnification and the length of the zoom group.**

**S5: Imaging quality evaluation of the continuous zoom microscope based on liquid lens**

The spot formed by the intersection of rays emitted from an object point and filling the entrance pupil is referred to as the spot diagram, which directly reflects the imaging quality. The root mean square (RMS) radius provides a quantitative measure of spot dispersion and serves as an indicator of image quality. It is calculated by squaring the distance of each ray from a reference point, averaging these values, and then taking the square root. When the spot diagram lies within the Airy disk, the system is considered to achieve diffraction-limited imaging.

Table S1 presents the RMS radius of the continuously zooming microscope objective at different magnifications. At all magnification levels, the RMS radii across the entire field of view remain smaller than the Airy disk radius, demonstrating that the designed system is capable of producing high-quality, diffraction-limited images.

**Table S1 RMS radius of the microscope objective at different magnifications (μm)**

| **Field of view**  **Magnification** | **10×** | **20×** | **30×** | **40×** | **50×** | **60×** | **70×** | **80×** | **90×** | **100×** |
| --- | --- | --- | --- | --- | --- | --- | --- | --- | --- | --- |
| **0.000 mm** | 0.328 | 0.489 | 0.412 | 0.371 | 0.352 | 0.422 | 0.415 | 0.563 | 0.546 | 0.367 |
| **2.828 mm** | 0.619 | 0.662 | 0.556 | 0.441 | 0.413 | 0.485 | 0.463 | 0.567 | 0.568 | 0.456 |
| **4.000 mm** | 0.799 | 0.731 | 0.606 | 0.558 | 0.533 | 0.626 | 0.585 | 0.654 | 0.633 | 0.605 |
| **Airy Spot Radius** | 0.694 | 0.676 | 0.649 | 0.631 | 0.616 | 0.603 | 0.554 | 0.573 | 0.571 | 0.456 |

According to the design specifications, the MTF (Modulation Transfer Function) curves of the system were obtained under visible wavelengths (0.486 μm, 0.587 μm, and 0.656 μm) at magnification levels of 10×, 20×, 40×, 60×, 80×, and 100×, as shown in the Fig. S6. The black curves represent the diffraction-limited MTF, while the blue, green, and red curves correspond to image heights of 0.000 mm, 2.828 mm, and 4.000 mm, respectively. Solid lines and dashed lines represent the MTF curves in the sagittal and tangential planes, respectively. From the plots, it can be observed that at all magnification levels and across different fields of view, the system MTF curves closely approach the diffraction limit. When MTF≥0.1, the spatial frequency exceeds 1300 lp (mm^-1^) for all magnification levels (10×, 20×, 40×, 60×, 80×, and 100×), indicating outstanding optical performance. The simulation results confirm that the proposed large-zoom-ratio, high-resolution continuous optical zoom objective lens consistently approaches diffraction-limited resolution across a wide range of magnifications, demonstrating the excellent imaging quality of the system.


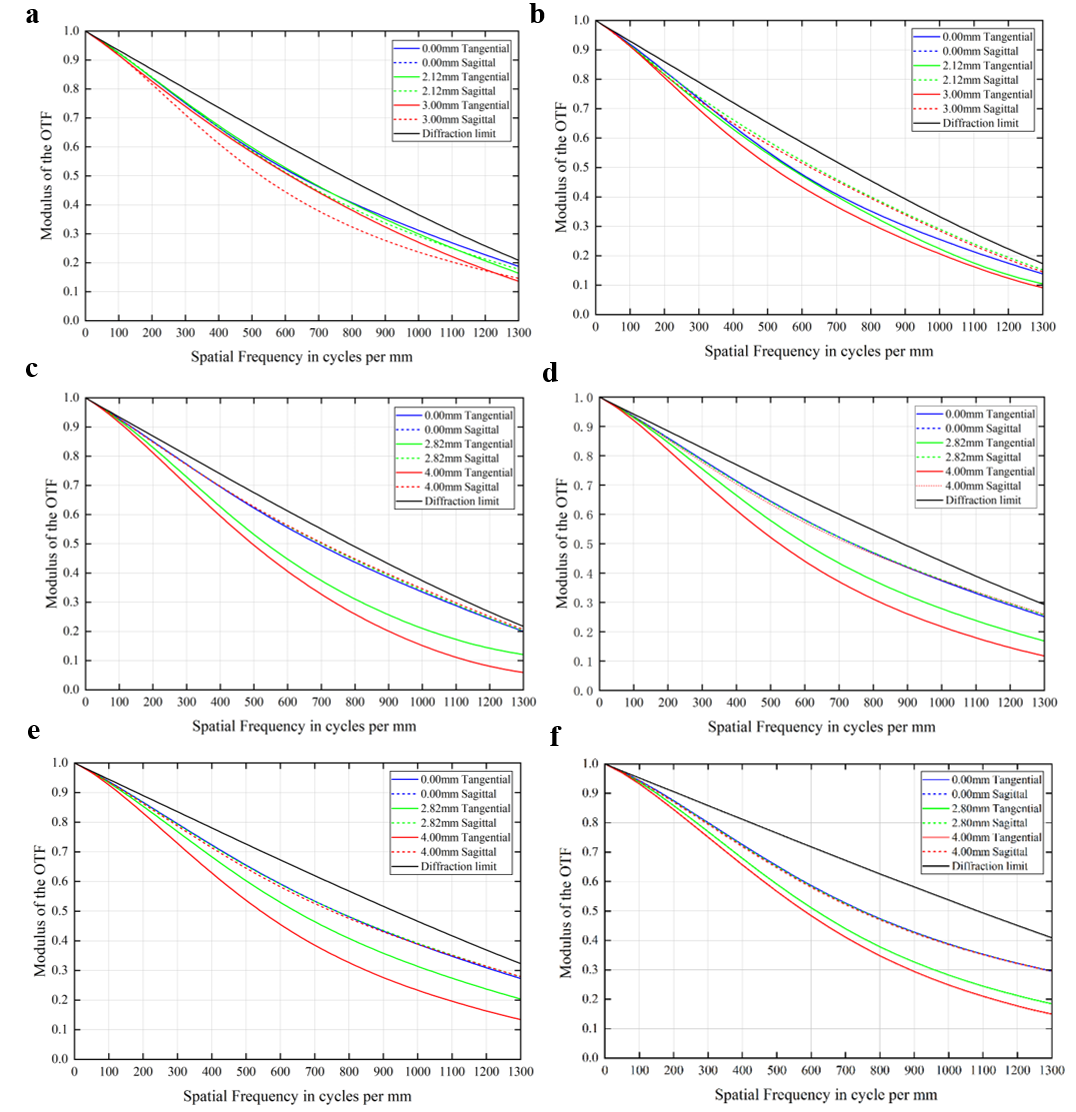


**Fig. S6 MTF of the zoom objective lens at different magnifications. a** MTF of magnification of 10×. **b** MTF of magnification of 20×. **c** MTF of magnification of 40×. **d** MTF of magnification of 60×. **e** MTF of magnification of 80×. **f** MTF of magnification of 100×.

Table S2 shows the analysis of aberration at different magnifications. To achieve high resolution, greater weight was assigned to the aberration optimization operands at 100× magnification during the optimization process. As a result, the aberration performance at 100× is significantly better than that at other magnification levels. In order to achieve a large zoom ratio, the minimum magnification was set to 10×. Thus, in the corresponding multi-configuration optimization, higher weight was assigned to the magnification operand at 10×, which led to relatively larger residual aberrations. For intermediate magnification levels, the conflict between the magnification operand and the aberration operand is relatively minor, resulting in comparatively better aberration correction. At low magnifications, however, this conflict is more pronounced, leading to relatively worse aberration optimization results. Nevertheless, the maximum residual aberration across all magnification levels is less than ±0.003 mm, which is negligible in practical imaging systems and can be considered as diffraction-limited performance. Under all these image quality evaluation criteria, it is demonstrated that the proposed continuous optical zoom microscope objective is capable of delivering high-quality imaging.

**Table S2 Simulation of image aberration of large zoom ratio continuous optical zoom objective lens（unit:** **μm）**

| **Aberration** | **Spherical aberration** | **Coma** | **Astigmatism** | **Field curvature** | **Distortion** | **Lateral chromatic aberration** | **Longitudinal chromatic aberration** |
| --- | --- | --- | --- | --- | --- | --- | --- |
| 10× | -0.043 | 0.016 | -0. 401 | 1.350 | 2.792 | -0.066 | -1.336 |
| 20× | 0.889 | 0. 293 | -0. 036 | 1.100 | 2.310 | 0. 220 | -0. 437 |
| 30× | 1.553 | 0. 106 | 0. 098 | 0. 549 | 1.915 | 0. 146 | 0. 115 |
| 40× | 1.305 | -0.004 | -0. 313 | 0.588 | 2.667 | -0.007 | 0. 473 |
| 50× | 1.460 | -0. 335 | -0. 292 | 0.414 | 1.846 | 0.017 | 0. 351 |
| 60× | 1.499 | -0. 317 | -0. 310 | 0.329 | 1.378 | -0.053 | 0. 427 |
| 70× | 1.295 | -0. 285 | -0. 242 | 0.239 | 0. 992 | -0. 157 | 0. 391 |
| 80× | 1.147 | -0. 281 | -0. 225 | 0.211 | 0. 824 | -0. 200 | 0. 375 |
| 90× | 1.719 | -0. 317 | -0.059 | 0.121 | 0. 343 | -0. 108 | 0. 287 |
| 100× | 0.053 | 0.003 | -0.059 | 0.145 | 2.861 | -0. 831 | 0. 681 |

It is worth noting that the analysis of phase aberration and chromatic aberration is based on optical simulation results derived from Zemax software. However, the actual situation is affected by manufacturing tolerances, resulting in inaccurate results. Therefore, it is difficult to obtain better aberration correction effects through traditional deconvolution-based algorithms.

**S6: Voltages applied to liquid lenses at different magnifications**

According to the optimized solution of the liquid lens curvature in Zemax, we obtain the voltages applied to each liquid lens at different magnifications. Fig. S7 shows the relationship between the magnification and the applied voltage. The magnification range is divided into high magnification area and low magnification area with a boundary of 40×. Within the low-magnification region (10× to 40×), changing the magnification has a significant impact on the system's magnification ratio. Therefore, the driving voltage variation range of each liquid lens is relatively large. In the medium and high magnification range (40× to 100×), changing the magnification has a relatively small impact on the system magnification ratio. Therefore, the voltage variation ranges of liquid lens-1, liquid lens-3 and liquid lens-4 are small, and the change of magnification is mainly regulated by other liquid lenses.

**
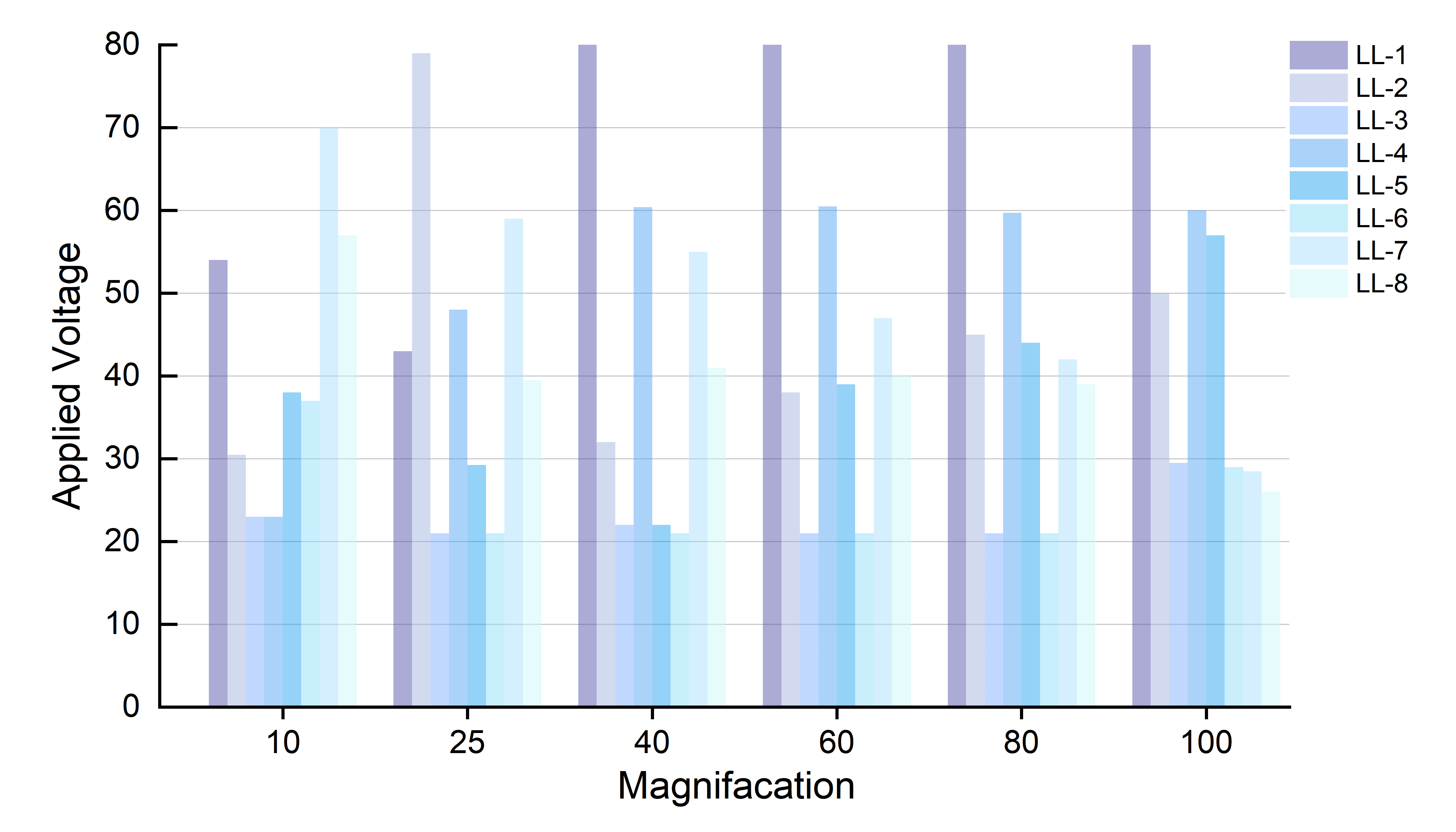
**

**Fig. S7 Voltages applied to liquid lenses at different magnifications. Voltage (V). (LL: Liquid lens)**

Through Zemax simulation of the system, the corresponding voltage control combinations for different zoom states can be obtained. During the continuous zoom process from 10× to 100×, some liquid lenses primarily undertake optical power adjustment, exhibiting significant changes in driving voltage, while others mainly serve for aberration correction and image plane position compensation, showing relatively small voltage variations. Table S3 shows the specific variation trends for each liquid lens.

**Table S3 Analysis of the trend of different liquid lens voltages with zoom ratio.**

| LL-1 | In the low-magnification range (10×-20×), the driving voltage exhibits a continuous decrease. Between 20× and 40×, the voltage rapidly increases to the maximum positive optical power and remains stable throughout the high-magnification range, primarily serving to adjust the focal length and regulate the primary optical power during magnification transitions. |
| --- | --- |
| LL-2 | In the low-magnification range (10×-20×), the driving voltage increases rapidly. Between 20× and 40×, the voltage decreases sharply, followed by a gradual increase in the high-magnification range (40×-100×). The functional role transitions from initial focusing to aberration compensation. |
| LL-3 | Across the entire magnification range, the driving voltage variation is minimal. This lens primarily functions for aberration correction and image plane stabilization, with only minor voltage adjustments required to maintain imaging stability. |
| LL-4 | In the low-magnification range, the driving voltage gradually increases, contributing significantly to focal length adjustment. Upon entering the high-magnification range, its role shifts toward aberration correction and image plane stabilization, with the voltage variation becoming less pronounced. |
| LL-5 | The driving voltage gradually decreases in the low-magnification range and then increases progressively in the high-magnification range, primarily to compensate for aberration changes and maintain image plane stability during high-magnification imaging. |
| LL-6 | In the low-magnification range (10×-30×), the driving voltage decreases and then stabilizes. In the high-magnification end range (90×-100×), the voltage gradually increases to compensate for aberrations and field curvature at long focal lengths. |
| LL-7 | The driving voltage exhibits a gradual decreasing trend across the entire magnification range, primarily contributing to the suppression of peripheral aberrations and the auxiliary balancing of the system’s overall optical power. |
| LL-8 | Similar to LL-7, the driving voltage decreases continuously throughout the magnification range, functioning cooperatively to optimize edge image quality and maintain the flatness of the image plane. |

Driven by electrowetting effect, the liquid lens offers a wide optical power range with high adjustment precision. In addition, the system incorporates eight liquid lenses, greatly enhancing the flexibility of focal length combination and adjustment. As a result, both in simulation and in practical operation, continuous zooming can be achieved at virtually any magnification. To further demonstrate the continuity of magnification adjustment, we also present optical simulation results and corresponding voltage settings for several non-specific intermediate magnifications, as shown in Figs. S8 and S9.


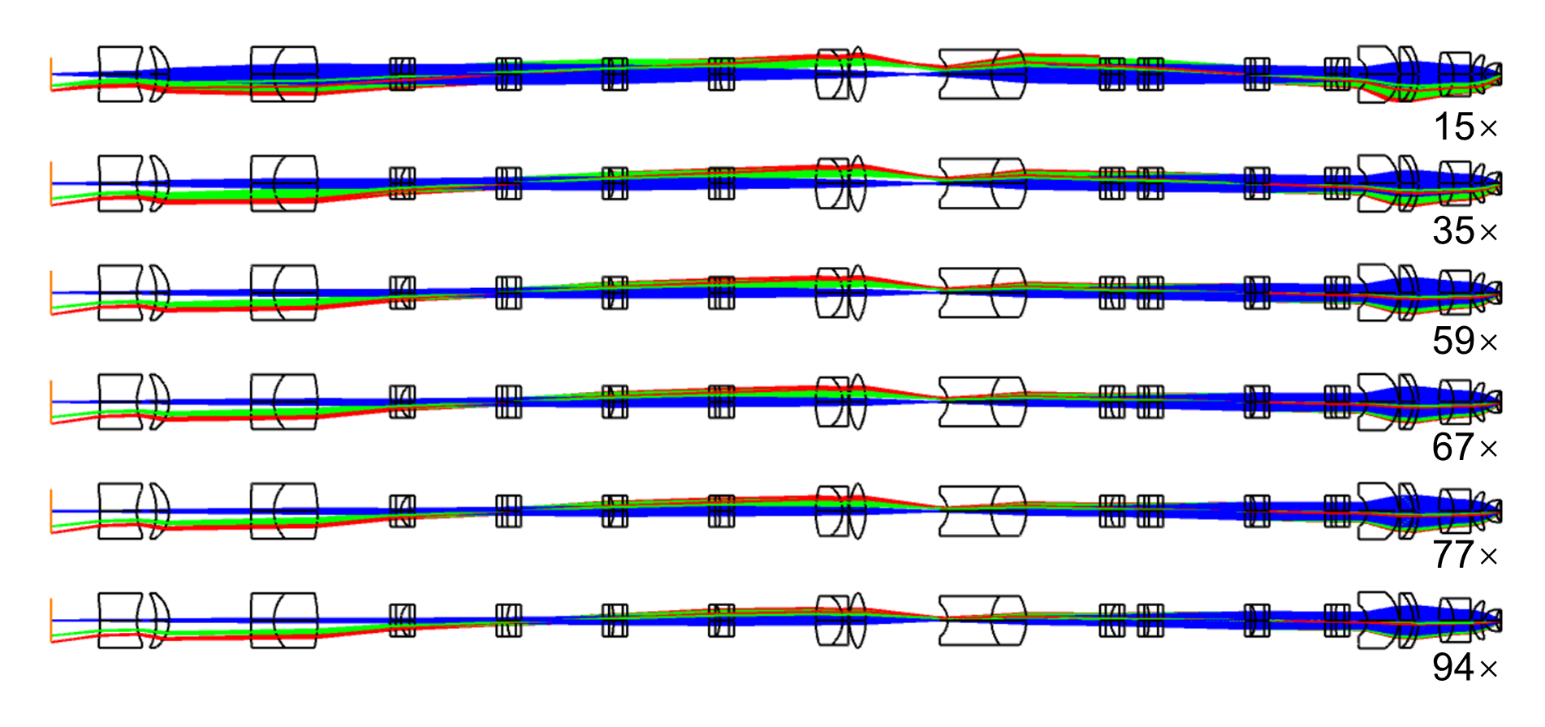


**Fig. S8 Simulation results of the zoom objective lens at the magnifications of 15×, 35×, 59×, 67×, 77×, and 94×.**


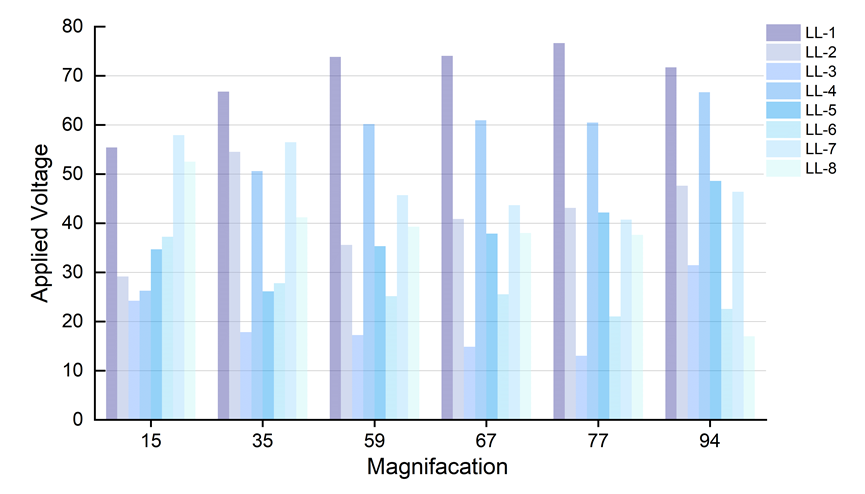


**Fig. S9 Voltages applied to liquid lenses at different magnifications. (LL: Liquid lens)**

For different types of liquid lenses, parameters such as size, clear aperture, driving voltage, focal length adjustment range, and control precision vary, necessitating targeted optimization in the design phase based on their specifications. Even for liquid lenses of the same model, minor variations may exist due to manufacturing tolerances. To ensure system accuracy, a voltage calibration process is performed prior to use. Specifically, we first obtained the optical focal length of the liquid lens at different magnifications based on simulation results and calculated the corresponding driving voltage. After applying the simulation results to the actual system, due to deviations from the simulation caused by manufacturing tolerances, fine-tuning was required to determine the initial optical focal length-voltage curve as a benchmark. For each liquid lens in the system, we used a lensmeter (COT-L800) to measure its optical focal length-voltage curve and compared it with the benchmark curve. Any differences were compensated by adjusting the driving voltage to ensure that the liquid lens used in the system can stably achieve the expected optical performance.

**S7: Additional description of the experiment**

**S7.1: Evaluation metrics for the quality of the reconstructed image**

The PSNR and structural similarity (SSIM) are used to evaluate the quality of the reconstructed image. The PSNR can be expressed as follows:

$$\text{PSNR}=20\cdot\lg\left(\frac{1}{\sqrt{\frac{1}{mn}\sum_{i=0}^{m-1} \sum_{j=0}^{n-1} \left\|X(i,j)-Y(i,j)\right\|^{2}}}\right)$$

where *X*(*i*, *j*) and *Y*(*i*, *j*) represent the object image and the reconstructed image with a size of *m*×*n*, respectively. The pixel values of the calculated image are taken in the range of 0 to 1. The SSIM can be expressed as follows:

$$\text{SSIM}=(\frac{2\mu_{\text{x}}\mu_{\text{y}}+c_{1}}{\mu_{\text{x}}^{2}+\mu_{\text{y}}^{2}+c_{1}})(\frac{2\sigma_{\text{xy}}+c_{2}}{\sigma_{\text{x}}^{2}+\sigma_{\text{y}}^{2}+c_{2}})$$

where *μ*_x_ and *μ*_y_ represent the mean values of the generated and the target image, respectively. *σ*_x_ and *σ*_y_ represent the standard deviation of the generated and the target image, respectively. *σ*_xy_ represents the covariance of the generated and the target image. *c*_1_ and *c*_2_ are two constant terms that are used to prevent the denominator from being zero.

**S7.2: Ablation experiment of the Content-Adaptive Learnable Wiener Deconvolution**

To validate the effectiveness of the CALW module, we conducted an ablation experiment by removing CALW from the 4DPSF-PDNet framework and comparing the results with a variant in which CALW was replaced by a non-learnable Wiener deconvolution method. As shown in Fig. S10, Degradation-guided Transformer (DGT) with Physical Degradation Feature Fusion (PDFF) effectively suppresses aberrations and restores image content. Meanwhile, the CALW based on PSF as a physical prior contributes to the recovery of fine image details. Compared to the non-learnable Wiener deconvolution, CALW achieves better restoration of edge details, demonstrating its superiority in handling degradation adaptively.


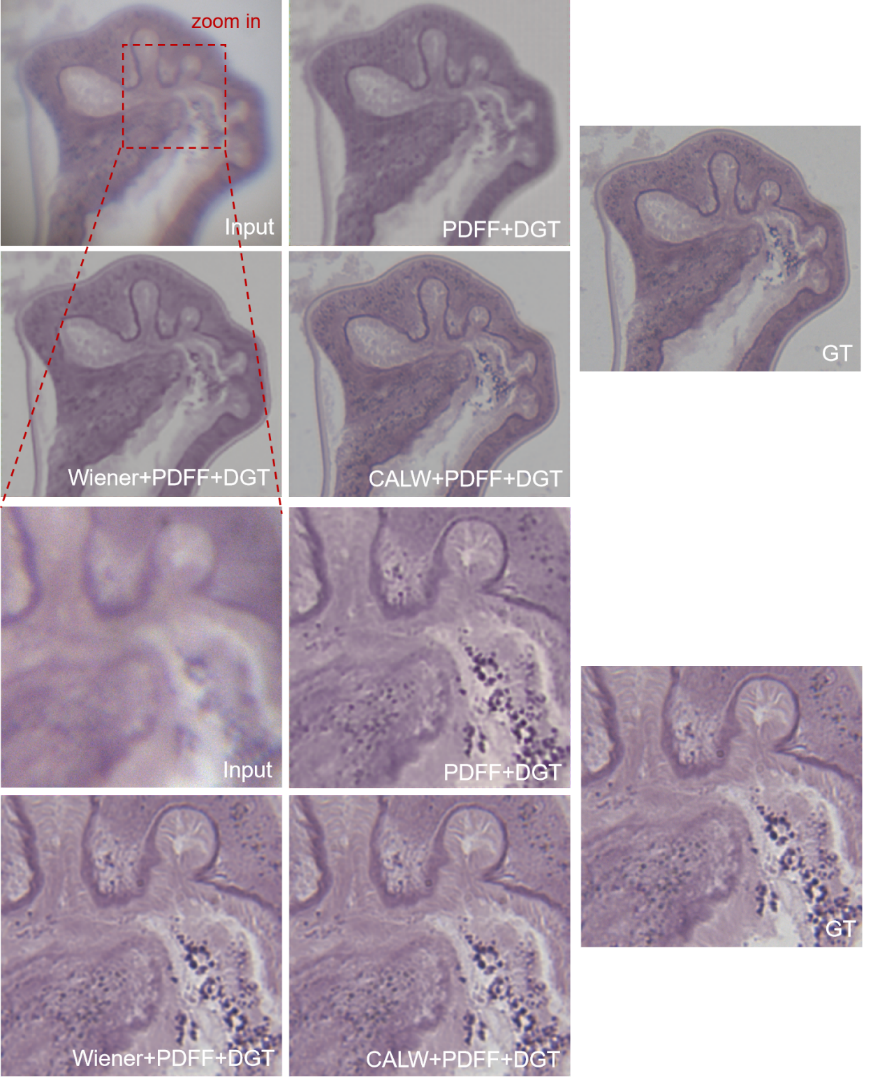


**Fig. S10 Ablation Experiment of the CALW and comparison with Non-Learnable Wiener Deconvolution on transverse section specimen of roundworm.**

**S7.3: Ablation experiment of the Degradation-guided Multi-Head Self-Attention mechanism**

To verify the effectiveness of our proposed DGMSA in adaptively correcting aberrations and restoring details using the PSF, we conducted ablation experiments. Without using the PDFF, we replaced the proposed DGMSA with window-based MSA (W-MSA) and global MSA (G-MSA). Both methods learn only from the image itself and lack the PSF as a guide for degradation information. As shown in Fig. S11, DGMSA in our DGT combines PDFF with the degradation information guidance of PSF, achieving better detail restoration effect than W-MSA and G-MSA while maintaining a low computational complexity.


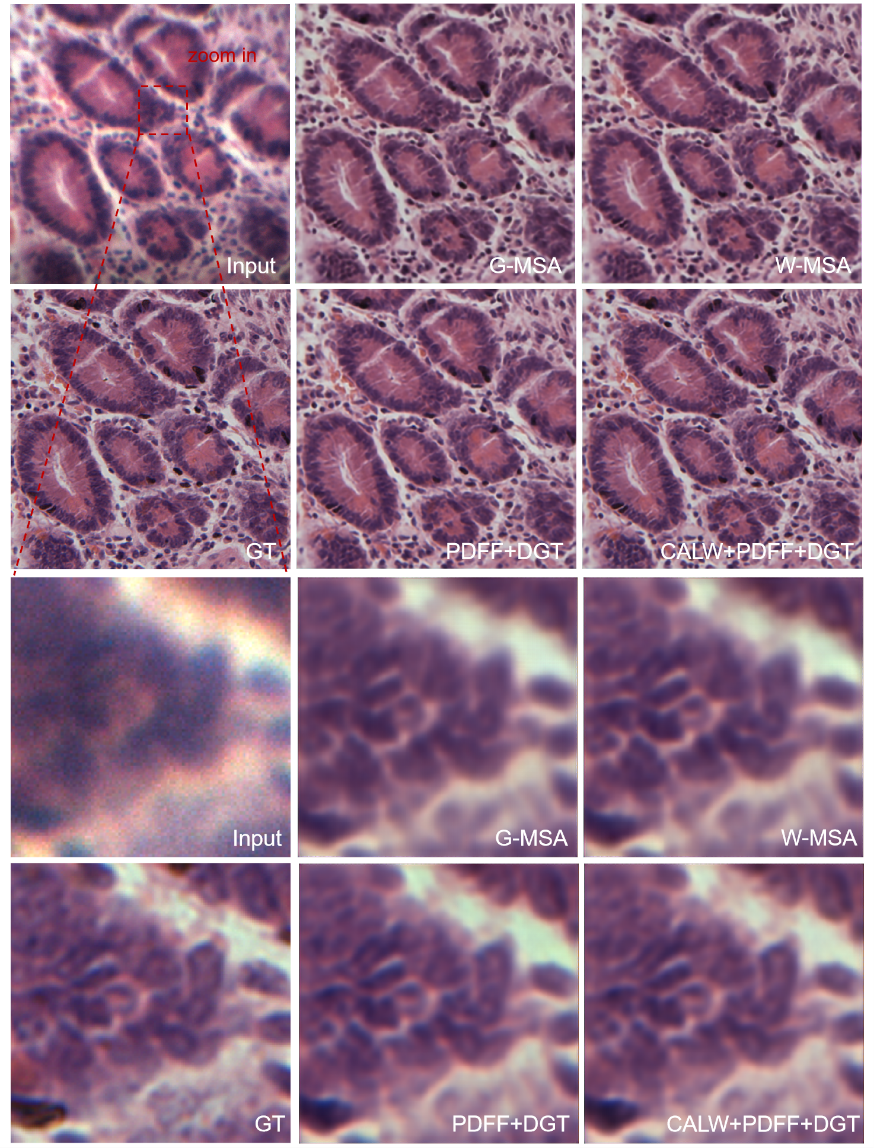


**Fig. S11** **Ablation Experiment of the DGMSA and comparison with window-based MSA (W-MSA) and global MSA (G-MSA) on chronic gastric ulcer biopsy sample.**

**S7.4: Experimental supplements on model effectiveness and generalization**

This paper tested samples from multiple species. Human samples included both internal tissues (small intestine slice, Fig. 3d) and surface tissues (skin sweat gland, Fig. 4b), as well as pathological specimens (chronic gastric ulcer sections, Fig. S11). Furthermore, the study was extended to other invertebrates (ascaris transverse sections, Figs. S10 and S12) and plant samples (leaves, Fig. 4a), demonstrating the method's broad effectiveness.

Furthermore, the model was trained on a comprehensive dataset of 68 different microscopic specimens. This dataset was intentionally broad, encompassing both plant and animal specimens, to ensure that the network could learn to correct aberrations across a wide range of textures and structures, not just those specific to a specific specimen type.


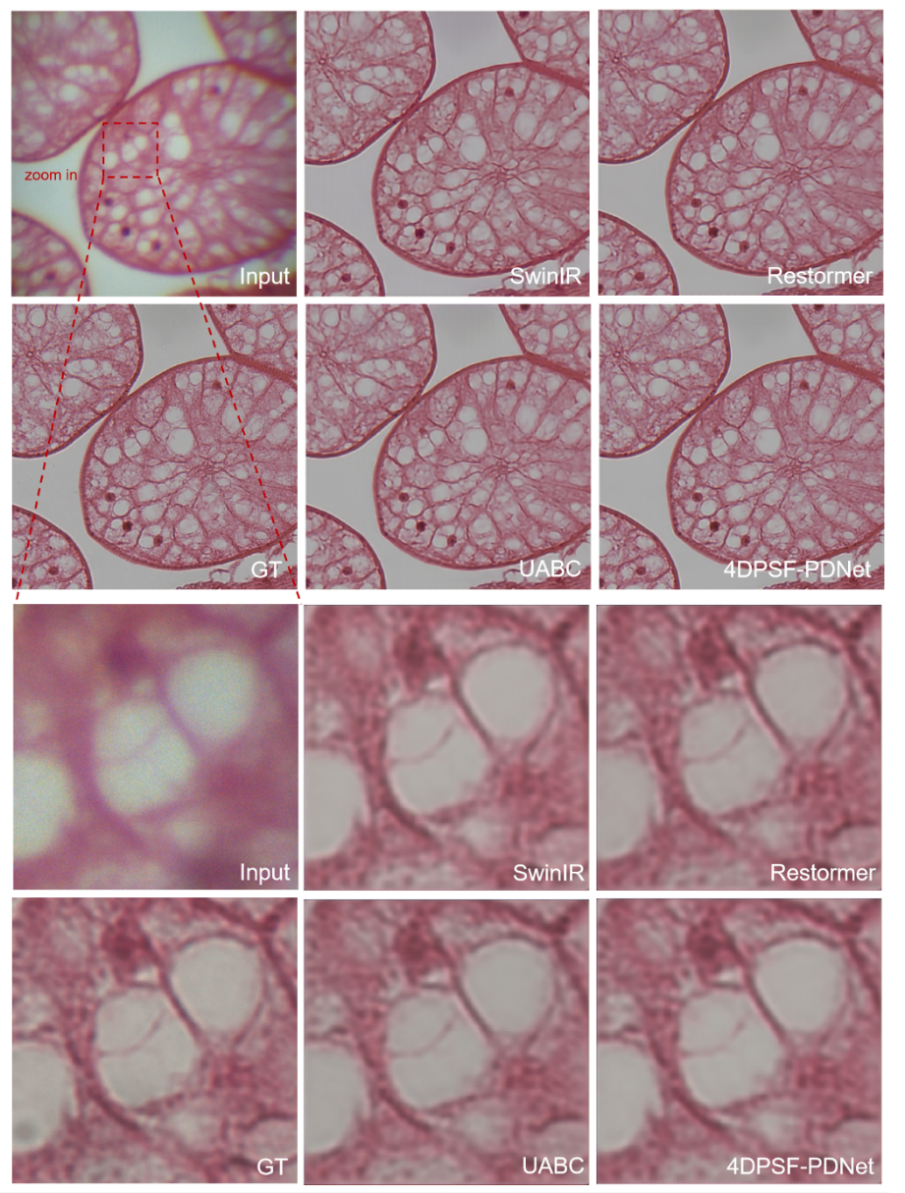


**Fig. S12** **Comparison of network processing results with other advanced models on the transverse section specimen of female ascaris.**

**References**

1. Pronina, V. et al. Microscopy image restoration with deep wiener-kolmogorov filters. In Computer Vision–ECCV 2020. Proc., Part XX 16, 185–201 (2020).
2. Qiao, C. et al. Evaluation and development of deep neural networks for image super-resolution in optical microscopy. Nat. Methods **18**, 194–202 (2021).
3. Zhu, J., Chen, X., He, K., LeCun, Y., & Liu, Z. Transformers without normalization. arXiv preprint arXiv:2503.10622 (2025).
